# Improving quality through process change: a scoping review of process improvement tools in cancer surgery

**DOI:** 10.1186/1471-2482-14-45

**Published:** 2014-07-19

**Authors:** Alice C Wei, David R Urbach, Katharine S Devitt, Meagan Wiebe, Oliver F Bathe, Robin S McLeod, Erin D Kennedy, Nancy N Baxter

**Affiliations:** 1 Princess Margaret Cancer Centre, University Health Network, Department of Surgery, University of Toronto, Toronto, ON, Canada; 2 Institute of Health Policy, Management and Evaluation, University of Toronto, Toronto, ON, Canada; 3 Department of Surgery and Oncology, University of Calgary, Calgary, AB, Canada; 4 Division of General Surgery, Mount Sinai Hospital, Toronto, ON, Canada; 5 Division of General Surgery, St. Michael’s Hospital, Toronto, ON, Canada

**Keywords:** Surgical procedures, Operative, Quality, Safety, Neoplasms, Scoping review

## Abstract

**Background:**

Surgery is a cornerstone of treatment for malignancy. However, significant variation has been reported in patterns and quality of cancer care for important health outcomes, including perioperative mortality. Surgical process improvement tools (SPITs) have been developed that focus on enhancing the processes of care at the point of care, as a means of quality improvement. This study describes SPITs and develops a conceptual framework by synthesizing the available literature on these novel quality improvement tools.

**Methods:**

A scoping review was conducted based on instruments developed for quality improvement in surgery. The search was executed on electronically indexed sources (MEDLINE, EMBASE, and the Cochrane library) from January 1990 to March 2011. Data were extracted, tabulated and reported thematically using a narrative synthesis approach. These results were used to develop a conceptual framework that describes and classifies SPITs.

**Results:**

232 articles were reviewed for data extraction and analysis. SPITs identified were classified into 3 groups: clinical mapping tools, structure communication tools and error reduction instruments. The dominant instrument reported were clinical mapping tools, including: clinical pathways (113, 48%), fast track (46, 20%) and enhanced recovery after surgery protocols (36, 15%). Outcomes reported included: length of stay (174, 75%), readmission rates (116, 50%), morbidity (116, 50%), mortality (104, 45%), and economic (60, 26%). Many gaps in the literature were recognized.

**Conclusion:**

We have developed a conceptual framework of SPITs and identified gaps in current knowledge. These results will guide the design and development of new quality instruments in surgery.

## Background

Surgery is a central modality in the treatment of cancer. The majority of patients with cancer will undergo a surgical procedure during the course of their treatment, for diagnostic, curative and/or palliative indications. For solid tumors, surgery is the cornerstone of curative treatment. As an example, for patients with colorectal cancer, one of the most common causes of cancer in high resource nations, resection is performed in 71-90% of patients. Surgery is the sole modality of treatment in up to 53% of patients [1-6].

Despite the important role of surgery in the care of patients with cancer, the quality of surgical care is not uniform. Significant variation in the patterns of care delivery and the quality of care has been reported, as reflected by clinically important differences in quality outcomes such as perioperative mortality and long-term survival [7-12]. For many procedures, perioperative mortality has a strong inverse relationship with case volume. Better outcomes are reported at high volume surgeons and/or institutions. The strength of the relationship between volume and outcomes is strongest for highly complex procedures such as cardiac surgery, and/or pancreatectomy [8,9]. In Ontario Canada, Simunovic et al. reported a 11.3% perioperative mortality rate in low volume hospitals compared to 3.4% in high volume hospitals [9] for pancreatectomy Similar trends have been demonstrated, to a lower magnitude, in most other cancer procedures including lung, breast and colorectal procedures [8,12-21].

The means by which increased institutional volumes affect outcome are complex and poorly understood. Khuri et al. have argued that procedure volume does not directly affect outcome and is likely a surrogate measure of implicit quality of the systems of care [8,10,13,18,21-30]. As a result, structural interventions are important but are insufficient to realize optimal quality targets. We believe that quality improvement interventions must move beyond strategies that focus solely on structural changes in health care delivery. We propose that gains in quality must include direct interventions at the point of care –interventions that improve the processes of care that are implemented at the time of care delivery.

The goal of this study was to identify and characterize the range of process improvement tools that have been developed in cancer surgery. We used scoping review methodology to synthesize and integrate the current literature. These data were used to develop a conceptual framework of this family of instruments and identify potential gaps in our knowledge about surgical process improvement instruments that warrant further evaluation for primary research and development.

## Methods

### Theoretical framework

There is currently no conceptual framework that integrates process improvement instruments in surgery into a single model. Within some individual areas (e.g. clinical pathways), conceptual frameworks have been described that model either the format and/or the intent of each individual tool and/or related instruments.

This scoping review was designed to identify surgical process improvement tools (SPITs) that shared the following properties:

1) Affect processes of care (i.e. practices involved in the actual giving and receiving of care)

2) Involve interventions that are delivered at the point of care (i.e. occur at the point in time when care is delivered)

3) Aim to codify, make explicit, standardize and/or operationalize processes of care

4) Incorporate current practices and/or introduce new practices into the content of the instrument

We considered that these criteria would map out a distinct group of quality improvement tools that shared a similar mechanism of action (process change) at a similar point in the health care cycle (point of care).

### Approach

We conducted a scoping review using various sources of data, and by consulting with quality improvement experts. A scoping review is appropriate for a topic that has not previously been thoroughly reviewed, or where literature may be sparse [31]. Its purpose is to evaluate the extent and nature of existing literature in areas where either insufficient evidence exists in order to conduct a systematic review or when synthesizing available evidence to begin describing a particular concept or phenomenon, and generate research questions where gaps in knowledge are revealed. This approach has been selected for our research question, as this is the first study (to our knowledge) that aims to integrate the disparate literature on SPITs into a single family of related quality improvement tools.

The methods are similar in rigor to a systematic review, but selection criteria are developed post hoc based on increasing familiarity with the literature, and detailed information is extracted to help readers contextualize the findings. The methods for extracting and describing eligible literature, which is likely to be diverse and include both qualitative and quantitative studies, was guided by Mays, who suggests that “narrative review” of information from various types of studies is appropriate for developing knowledge at an early stage in policy development [31,32]. This involves thematic rather than statistical analysis of information to explore concepts and relationships, and consultation with experts to further contextualize the findings so that they are applicable to stakeholders.

### Data collection

#### Sources

Several indexed, non-indexed and expert sources were consulted. Search strategies were designed specific to the sources, and combined concepts reflecting surgery and tools or communication or quality or safety. Various stakeholders representing physicians (Canadian Association of General Surgeons), institutions (University Health Network hospital administration) and policy level stakeholders (Cancer Care Ontario) were engaged to review the initial selection criteria, in order to identify gaps in the search strategy and to provide additional concepts that informed the search strategy. Searches of indexed sources were executed from January 1990 to March 2011, the period during which concepts of quality improvement in surgery gained traction. These databases included MEDLINE (North American), the Cochrane Library (systematic reviews, trials) and EMBASE (European). In addition, to ensure that all relevant literature is captured, we hand searched the references of eligible studies. It was anticipated that any seminal papers published prior to 1990 would be retrieved from scanning the references of extracted studies.

#### Selection criteria

Preliminary selection criteria include quantitative (meta-analyses, systemic reviews, surveys, observational studies, randomized trials) or qualitative (reviews/conceptual analyses, interviews, focus groups) studies published in English language peer-reviewed journals from January 1990 to March 2011 and limited to humans, provided that they focused on developing or evaluating SPITs (as defined above), and provided sufficient detail to extract study design and findings. Studies were deemed ineligible if they evaluated processes of care solely for the purposes of performance management, such as studies that report results of quality indicators. Abstracts, addresses, autobiographies, bibliographies, biographies, comments, editorials, letters and newspaper articles, were excluded

#### Selection methods

Titles and abstracts of the search results and select articles/tools were screened for inclusion based on eligibility criteria. If more than one publication described a single study, only most recent was included. Once the full text articles and tools were retrieved, 2 abstractors independently extracted data. The initial selection criteria were refined based on the information extracted during by the initial scope of the literature. The updated selection criteria were utilized to identify additional eligible items for inclusion in the review.

### Data analysis

#### Data extraction

A data extraction form was developed based on the conceptual framework. Notable qualitative details were identified on the data extraction form. Quality criteria relevant to study design was developed using criterion based approach [33-37] After meeting to compare the data extracted from all selected studies, differences in opinion between the abstractors were resolved through consensus. Extracted data was tabulated and presented thematically.

#### Data analysis and interpretation

The total number of eligible and included studies was reported, along with reasons for exclusion. Contextual information related to instrument design were examined thematically according to May’s narrative synthesis approach [32]. Qualitative content that was highlighted were reviewed in light of the proposed conceptual framework. Revisions were made to the conceptual framework, as guided by the nature and content of the data extracted, in order to clarify emerging concepts. Tabulated findings were examined to discuss the instrument type, quantity, design, study quality, clinical applications and relevant outcomes.

## Results

The aim of this project was to examine the development of SPITs in the area of cancer surgery. But, upon initial scope, we found that studies that focused on SPITs for cancer surgery was very limited. Thus, we modified our search strategy to include all areas of surgery in order to obtain a fuller view of the current literature.

**Figure 1:**
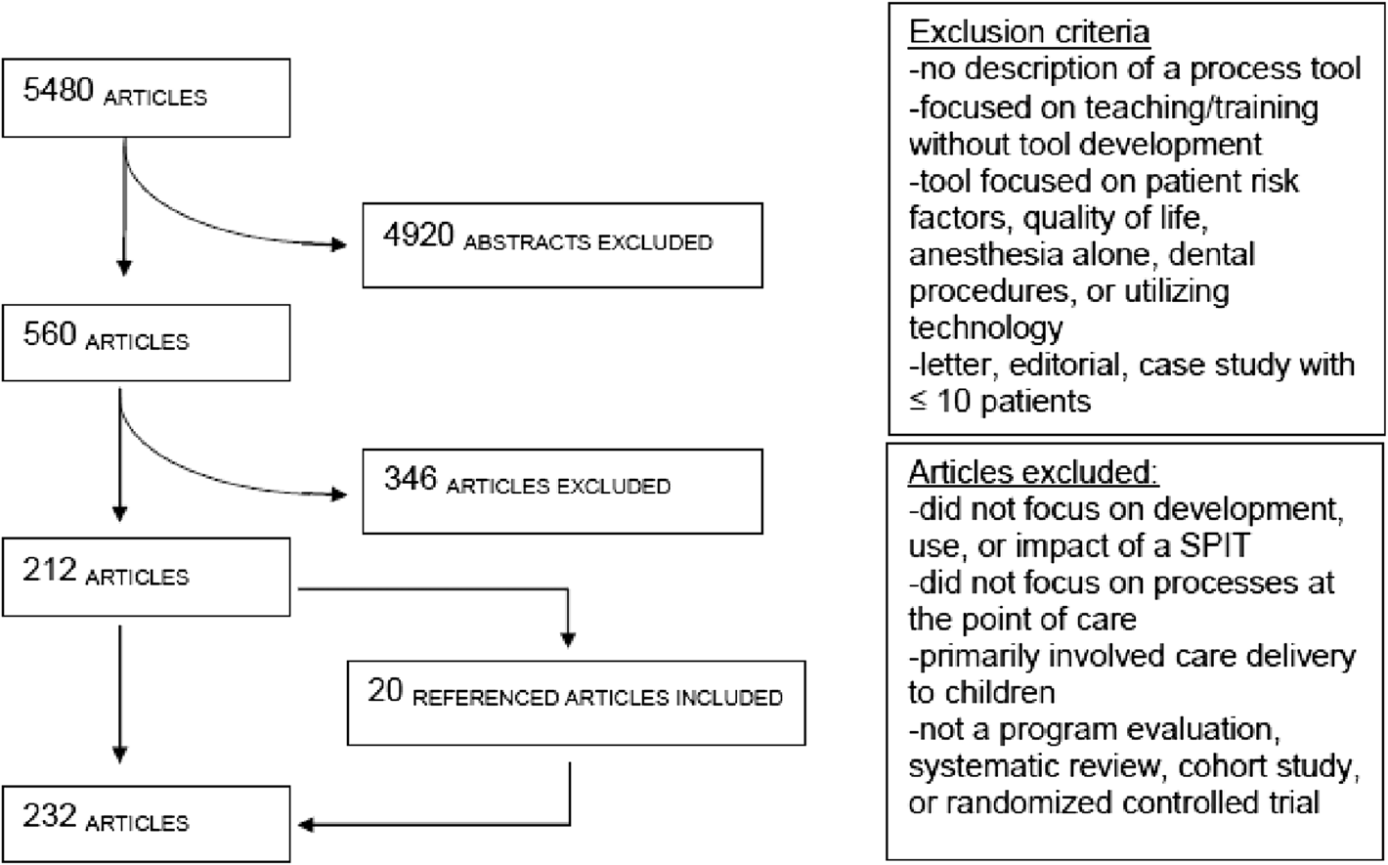
Scoping review search results.

The final search criteria extracted 7192 articles. 5480 remained after duplicates were removed, 4920 articles were excluded based on criteria selected for the abstract review (Figure 1). Abstracts were excluded if: they reported clinical/medical effectiveness without description of a process tool; concluded that a SPIT is needed without description of development, implementation, or evaluation; focused on teaching or training of health professionals or trainees without tool development; focused on instruments to identify patient risk factors, clinical conditions, quality of life, or with regard to preoperative anesthesia alone, dental procedures, or technology/computer tools; if the publication was a letter, editorial, case study with less than 10 patients, or not the most recent version of the research project. The remaining 560 articles were fully reviewed for eligibility. Of these, 212 articles were determined to meet all inclusion criteria and none of the exclusion criteria. Twenty additional articles were identified as relevant to this research project after hand searching the references of the eligible articles. Therefore, a total of 232 articles were reviewed for data extraction and analysis (Additional file 1: Table S1).

The quality of these studies was assessed using a criterion approach for the presence of: detailed inclusion and exclusion criteria, description of control group, tool compliance, whether primary outcomes were explicitly described and objective applied, and assessment or discussion of SPIT sustainability. Inclusion and exclusion criteria were only reported in 120 out of 219 articles (55%). When applicable, control groups were described in 88 out of 136 of the articles (65%). Tool compliance was reported in 59 out of 226 articles (26%). Primary outcomes were explicitly described in 192 out of 228 articles (84%) and the outcome criteria were considered objectively applied in 138 out of 223 articles (62%). Sustainability of the SPIT was only mentioned in 6 (3%) articles (Table 1).

**Table 1:** Quality assessment of SPITs articles

**Criterion**	**Explicit inclusion/exclusion criteria described**		**Controls described**		**Tool compliance reported**		**Primary outcomes explicitly described**		**Primary outcome criteria objectively applied**		**Sustainability of tool evaluated**	
	**N**	**%**	**N**	**%**	**N**	**%**	**N**	**%**	**N**	**%**	**N**	**%**
Present	120	55	88	65	59	26	192	84	138	62	6	3
Absent	99	45	48	35	167	74	36	16	41	18	226	94
Unknown	0	0	0	0	0	0	0	0	44	20	0	0
Not applicable	13	6	96	41	6	3	4	2	9	4	0	0

The majority of these studies were designed as either an interventional (115, 49%) or observational (72, 31%) assessment of the SPITs. Only 12 (5%) studies were randomized controlled trials (Table 2). The majority of articles study objectives were to: evaluate (120, 52%), implement (105, 45%), and/or describe (91, 39%) SPITs. Only a few articles compared two or more SPITs (10, 4%) or discussed SPIT development (42, 18%) (Table 2). The majority of these studies were completed at academic centers (158, 68%) and were located in the USA (113, 48%) or Europe (55, 24%).

**Table 2:** Study design and objectives of SPITs articles

**Study design**	**N**	**% **	** Study objectives**	**N**	**%**
Interventional	115	49	Tool evaluation	120	52
Observational	72	31	Tool implementation	105	45
Program evaluation	20	9	Tool description	91	39
Randomized controlled trial	12	5	Tool development	42	18
Systematic review	9	4	Tool comparison	10	4
Qualitative	3	1			
Survey	1	0			

Only 67 (29%) of the studies evaluated SPITs in the setting of cancer surgery. The majority of studies were within the colorectal (53, 23%), abdominal- non-colorectal (29, 12%), orthopedic (29, 12%), vascular (27, 12%), or cardiac surgical setting (27, 12%).

The most common SPITs were clinical pathways (113, 48%), fast-track protocols (46, 20%), and enhanced recovery after surgery (ERAS) protocols (36, 15%). Structured communication tools (17, 7%), checklists (16, 7%), patient care planning/management (14, 6%), preparatory pause (7, 3%) and patient safety (6, 3%) were less frequently reported (Table 3). Outcomes evaluated included: length of stay (174, 75%), readmission rates (116, 50%), morbidity (116, 50%), mortality (104, 44%) and economic impacts (60, 26%).

**Table 3:** Type of SPITs reported in articles

**Surgical process improvement tools (SPITs)**	**N**	**%**
*Clinical mapping instruments*		
Clinical pathway	113	48
Fast track protocol	46	20
Enhanced recovery after surgery protocols	36	15
*Structured communication instruments*		
Structured communication tool	17	7
Checklist	16	7
Preparatory pause	7	3
*Error reduction strategies*		
Patient care planning/management	14	6
Patient safety	6	3

Data were extracted for statistically significant outcomes. In articles that assessed these outcomes, length of stay (146, 84%) and economic impacts (53, 88%) were frequently reported as decreasing after implementation of the SPIT. When reported, SPIT implementation appeared to have no significant effect on morbidity (69, 59%), mortality (76, 73%) or readmission rates (86, 74%) (Table 4).

**Table 4:** Impact of SPIT for reported outcomes

**Outcomes**	**Increased**		**Decreased**		**No significant change**		**Not reported**	
	**N**	**%**	**N**	**%**	**N**	**%**	**N**	**%**
Length of stay	0	0		146	84	17	10	11	6
Readmission rates	8	7		8	7	86	74	14	12
Morbidity	1	1		27	23	69	59	19	16
Mortality	0	0		14	13	76	73	14	13
Economic	0	0		53	88	3	5	3	5

## Discussion

We report that the literature in the area of SPITs is presently under-investigated with only a handful of high quality studies. Only 232 relevant studies were identified; a mere 67 (29%) studies focused on cancer surgery. At present the quality of literature in this area is low. Nevertheless, this preliminary data suggests that these instruments may be capable of effecting important quality improvements.

An important feature of the studies identified is that patient-centered outcomes, such as mortality and morbidity, were not the primary focus in the majority of the studies; they were reported as secondary outcomes. The vast majority of studies focused on length of stay, cost containment and improved efficiencies of care as primary study endpoints. These data indicate that SPITs can reduce length of stay without negatively affecting patient safety (i.e. morbidity, mortality or readmission rates). Since patient-centered outcomes were only secondary endpoints, most studies were underpowered to fully evaluate these outcomes. Thus future research will be required to clarify whether SPITs has a positive impact on the important patient-centered outcomes such as morbidity, mortality and/or cancer survival.

**Figure 2:**
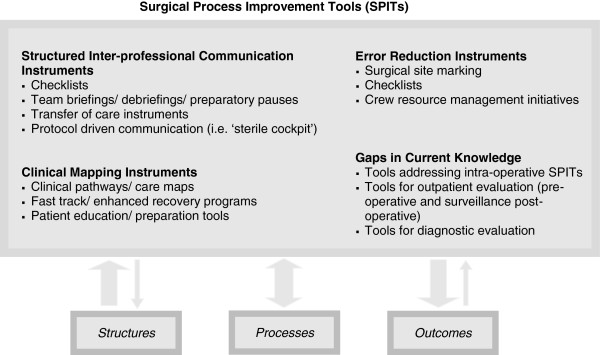
SPITs conceptual framework.

Our study reveals important gaps in knowledge within this area. Notably, few if any SPITs have been developed for intraoperative processes of care. It is peculiar that there were no SPITs that addressed any of the technical components of a procedure. Another knowledge gap included a paucity of SPITs that addressed the preoperative evaluation or the postoperative surveillance of cancer patients.A conceptual framework of SPITs was developed based on the results of this scoping review. We describe a group of quality improvement tools that change the processes of care, at the point of care. We grouped SPITs into 3 categories of tools: structured communication tools, clinical mapping tools and error reduction tools (Figure 2). These categories describe the major properties of the SPITs and reflect the main mechanism by which they can improve quality of care. Since these tools can have multiple mechanisms of action, they may have properties that overlap into more than one category. Gaps in current knowledge were also incorporated into the framework to direct areas for future research.

Structured communication tools include instruments such as: perioperative checklists, preparatory pauses, and team briefings. These instruments focus on operationalizing the steps of team communication and standardizing information exchange in specific settings [38-47]. The most common example in clinical use is the preoperative checklist - is primarily a structured communication tool, but elements of the checklist may involve verification of essential safety steps. Thus through its design and content a checklist also has error checking properties [39,40,48,49].

Clinical mapping tools are multi-disciplinary structured care plans that describe the timing and actions required by providers in order for the patient to meet target goals in a timely manner [50-53]. Clinical pathways are multi-disciplinary care maps that usually have a time dependent inventory of action for a specific episode of care. They have been widely implemented to improve patient efficiencies, reduce length of stay and economic costs through decreased variation in care delivery and early identification of outliers [50-57]. Quality of care has often been a secondary consideration for these instruments [55-60].

Some clinical mapping tools, such as enhanced recovery strategies and fast track protocols, emphasize pharmacologic changes or modification in current practices in order to facilitate early recovery (e.g. modifying perioperative pain control to facilitate early mobilization, early enteral feeding, etc.). Standardization of processes is a by-product rather than a primary goal of enhanced recovery strategies.

Error reduction instruments are developed to reduce the incidence of preventable errors such as wrong site surgery and/or errors in drug administration [44,46,47,61,62]. Often these tools are targeted to eliminate ‘never’ events such as wrong site/wrong patient surgery.

We postulate that SPITs can be described as a unique family of quality improvement interventions. They can be an important method of closing the knowledge-to-action gap between evidence and practice. This family of process improvement tools target process change as a means to improve quality. To date, most have focused on surrogate outcomes such as length of stay, cost, or patient safety, rather than any direct measure of quality of care such as perioperative morbidity, mortality and/or long term survival or appropriateness of care. Thus, the end effect of these tools on quality is not well understood. Further the relationship, relative merits and deficits of each type of SPIT, and areas of mutual overlap are not known. Despite the limited knowledge to date, we believe this family of quality improvement tools is important because they target processes of care and hold the potential to embed evidence-based quality improvement at the point of care. Further information is required about the relationship between this family of quality improvement instruments with each other, and with important quality outcomes.

There are limitations in this scoping review. Our study focused on SPITs instruments used in the immediate perioperative period. This was emphasized in our search strategy that required the term ‘surgery’ as a key element. As a result, it is possible that SPITs used during the early diagnostic and long-term surveillance period may have been missed. For patients with cancer, surgery usually makes up a small part of their cancer therapy. Diagnosis and surveillance activities may not involve the surgeon and therefore SPITs involving these activities may be beyond the scope of this study. Nevertheless, we would have expected that hand searching of articles reference would have at least key articles of note involving diagnosis and/or surveillance related SPITs. No substantial diagnostic or surveillance tools were identified in our scope. As a result, we believe that this area remains an important gap, requiring further research.

## Conclusions

SPITs are innovative knowledge instruments that can improve quality of health care by optimizing the processes of care delivery. They can directly influence components of care delivered, standardize and coordinate elements of care, and enhance uptake of best evidence. Importantly we sought to describe a group of tools that work at the point-of-care. In this way, SPITs can change the processes of care directly. This mechanism differs from other knowledge translation strategies such as continuing medical education strategies, which change behavior in more indirect ways. We believe that the limitations in the current knowledge provide opportunities for new research.

At present these instruments are implemented in an isolated fashion, without the benefit of a unified agenda of quality improvement. We suggest that further development and research into SPITs will demonstrate an important role these tools can play in high quality surgical care. The results of this study enrich our understanding of these types of knowledge products and unify them into a family of related tools. We anticipate that the results of this study will guide the design and development of new SPIT instruments for use with patients undergoing cancer surgery.

## Abbreviations

SPITs: Surgical process improvement tools; ERAS: Enhanced recovery after surgery.

## Competing interests

The authors declare that they have no competing interests.

## Authors’ contributions

ACW, NNB, DRU participated in study conception and design. OFB, RSM, BRT, NNB, EDK, and DRU refined and developed the primary search strategy. ACW, KD, MW were involved in drafting and revising the manuscript. ACW, KD, MW performed data collection and analysis. ACW, DRU, NNB, EDK provided critical revisions to the manuscript. ACW provided supervision of all aspects of the protocol. All authors read and approved the final manuscript.

## Pre-publication history

The pre-publication history for this paper can be accessed here:

http://www.biomedcentral.com/1471-2482/14/45/prepub

## Supplementary Material

Additional file 1: Table S1 Summary of articles from scoping review of SPITs. Click here for file
