# Supplementary material for: Improving quality through process change: a scoping review of process improvement tools in cancer surgery
Source: BMC Surg. 2014 Jul 19;14:45. doi: 10.1186/1471-2482-14-45 (PMC4112620; doi:10.1186/1471-2482-14-45)
Supplement: Additional file 1: Table S1 — Summary of articles from scoping review of SPITs. [file 1471-2482-14-45-S1.docx]

| **Author (Year)** | **Type of study** | **Surgical site** | **Cancer related** | **Type of tool** | **Study objective for SPITs** | **# of patients** | **Length of stay** | | **Morbidity** | **Readmission rate** | | **Mortality** | | **Economic outcomes** | |
| --- | --- | --- | --- | --- | --- | --- | --- | --- | --- | --- | --- | --- | --- | --- | --- |
| Aboulian et al (2010)^1^ | Observational | colorectal | Yes | Clinical pathway | Implementation | 27 |  |  | |  |  | | No | |  |
| Ad et al(2010)^2^ | Observational | cardiac | No | Clinical pathway | Development Implementation Evaluation | 391 | - | - | | - | - | | No | |  |
| Aguilar-Nasciment et al (2010)^3^ | Interventional | abdominal | No | Clinical pathway | Implementation Description | 114 |  |  | | - |  | | No | |  |
| Al Chalabi et al (2010)^4^ | Interventional | colorectal | Yes | Enhanced recover after surgery | Evaluation | 73 |  |  | |  |  | | No | |  |
| Altpeter et al (2007)^5^ | Program Evaluation | multi-procedure | No | Preparatory pause | Development Implementation Evaluation | 290 | - | - | | - | - | | No | |  |
| Andersen et al (2005)^6^ | Observational | colorectal | No | Fast-track protocol | Implementation | 32 |  |  | |  | - | | No | |  |
| Anderson et al (2003)^7^ | Randomized controlled trial | colorectal | No | Enhanced recover after surgery | Implementation | 25 |  |  | |  |  | | No | |  |

Additional file 1: Table S1: Summary of articles from scoping review of SPITs

**LEGEND**

- Not reported or not available

Decreased with tool

Increased with tool

No significant change

| Archer (1997)^8^ | Interventional | colorectal | No | Clinical pathway | Evaluation | 24 | |  | |  | |  | |  | | Yes | |  |
| --- | --- | --- | --- | --- | --- | --- | --- | --- | --- | --- | --- | --- | --- | --- | --- | --- | --- | --- |
| Austin Health Post-Operative Surveillance Team (POST) Investigators (2010)^9^ | Observational | multi-procedure | No | Checklist / Patient care planning | Development Description | 831 | | - | | - | | - | | - | | No | |  |
| Back et al (1997)^10^ | Interventional | vascular | No | Clinical pathway | Development Implementation Evaluation | 102 | |  | |  | |  | |  | | Yes | |  |
| Backster et al (2007)^11^ | Observational | multi-procedure | No | Checklist / Preparatory pause | Evaluation | 167 | | - | | - | | - | | - | | Yes | |  |
| Baird et al (2010)^12^ | Interventional | colorectal | No | Fast-track protocol | Evaluation | 200 | |  | |  | |  | | - | | No | |  |
| Baker et al (1999)^13^ | Program Evaluation | orthopedic | No | Clinical pathway | Development Implementation Description | 600 | |  | | - | | - | | - | | No | |  |
| Balzano et al (2008)^14^ | Interventional | abdominal | Yes | Enhanced recover after surgery | Description Comparison | 504 | |  | |  | |  | |  | | No | |  |
| Basse (2000)^15^ | Observational | colorectal | No | Fast-track protocol | Implementation Description | 60 | |  | |  | |  | |  | | No | |  |
| Basse et al (2002)^16^ | Interventional | colorectal | No | Enhanced recover after surgery | Implementation Description | 28 | |  | |  | |  | | - | | No | |  |
| Basse et al (2002)^17^ | Observational | colorectal | No | Enhanced recover after surgery | Implementation | 27 | |  | | - | | - | | - | | No | |  |
| Basse et al (2004)^18^ | Interventional | colorectal | No | Fast-track protocol | Evaluation | 260 | |  | |  | |  | |  | | No | |  |
| Beaupre et al (2006)^19^ | Interventional | orthopedic | No | Clinical pathway | Implementation Description Evaluation | 1341 | |  | |  | |  | |  | | Yes | |  |
| Becker et al (1997)^20^ | Interventional | urology | No | Clinical pathway | Development Implementation Evaluation | 307 | |  | |  | | - | | - | | Yes | |  |
| Berger et al (2006)^21^ | Observational | orthopedic | No | Clinical pathway | Implementation | 100 | | - | |  | |  | |  | | No | |  |
| Berger et al (2009)^22^ | Observational | orthopedic | No | Fast-track protocol | Description Evaluation | 150 | | - | |  | |  | | - | | No | |  |
| Berger et al (2009)^23^ | Observational | orthopedic | No | Fast-track protocol | Description Evaluation | 111 | | - | | - | |  | |  | | No | |  |
| Berry et al (2009)^24^ | Observational | cardiac | No | Clinical pathway | Development Implementation Description | 254 | |  | | - | |  | |  | | Yes | |  |
| Bleakley et al (2006)^25^ | Interventional | multi-procedure | No | Structured communication tool | Implementation | - | | - | | - | | - | | - | | No | |  |
| Braumann et al (2009)^26^ | Observational | colorectal | Yes | Enhanced recover after surgery | Evaluation | 748 | | - | | - | | - | | - | | No | |  |
| Broder et al (2002)^27^ | Interventional | gynecology | No | Clinical pathway | Evaluation | - | |  | | - | | - | | - | | No | |  |
| Brown et al (2001)^28^ | Qualitative | multi-procedure | No | Patient safety / Checklist | Development Description | 3600 | | - | | - | | - | | - | | No | |  |
| Brustia et al (2007)^29^ | Observational | vascular | No | Fast-track protocol | Evaluation | 323 | |  | | - | | - | | - | | No | |  |
| Bryan et al (2002)^30^ | Program Evaluation | endocrine | Yes | Patient care planning | Description | - | | - | | - | | - | | - | | No | |  |
| Buzink et al (2010)^31^ | Program Evaluation | abdominal | No | Checklist | Evaluation | 45 | | - | | - | | - | | - | | No | |  |
| Cabello et al (1998)^32^ | Interventional | abdominal | No | Clinical pathway | Development | 116 | |  | | - | | - | | - | | No | |  |
| Calland et al (2001)^33^ | Interventional | abdominal | No | Clinical pathway | Description Evaluation | 385 | |  | | - | |  | | - | | Yes | |  |
| Calligaro et al (1995)^34^ | Interventional | vascular | No | Clinical pathway | Evaluation | 322 | |  | | - | |  | |  | | Yes | |  |
| Calligaro et al (2004)^35^ | Interventional | vascular | No | Clinical pathway | Comparison | 109 | |  | |  | |  | |  | | Yes | |  |
| Carli et al (2009)^36^ | Observational | colorectal | Yes | Enhanced recover after surgery | Development Implementation | 25 | |  | |  | |  | | - | | No | |  |
| Carter et al (2010)^37^ | Observational | gynecology | Yes | Fast-track protocol | Implementation Description | 72 | |  | |  | |  | | - | | No | |  |
| Cayir et al (2007)^38^ | Interventional | urology | No | Clinical pathway / Patient care planning | Development Implementation Description | 32 | |  | | - | | - | | - | | No | |  |
| Cerfolio et al (2001)^39^ | Observational | thoracic | No | Fast-track protocol | Implementation | 500 | |  | |  | |  | |  | | No | |  |
| Cerfolio et al (2004)^40^ | Observational | abdominal | No | Fast-track protocol | Description Evaluation | 90 | |  | |  | | - | |  | | No | |  |
| Chalian et al (2002)^41^ | Interventional | ENT | Yes | Clinical pathway | Implementation | 51 | | - | | - | | - | | - | | No | |  |
| Chang et al (1999)^42^ | Interventional | urology | No | Clinical pathway | Implementation | 2661 | |  | | - | |  | |  | | Yes | |  |
| Chang et al (2003)^43^ | Interventional | urology | Yes | Clinical pathway | Evaluation | 124 | |  | |  | |  | |  | | Yes | |  |
| Chen et al (2000)^44^ | Interventional | ENT | Yes | Clinical pathway | Implementation Description | 190 | |  | | - | | - | | - | | Yes | |  |
| Claridge et al (2000)^45^ | Observational | abdominal | No | Patient care planning | Development Implementation Evaluation | 203 | |  | | - | | - | |  | | No | |  |
| Clark et al (1999)^46^ | Observational | cardiac | No | Clinical pathway / Patient care planning | Development Implementation | - | |  | | - | | - | | - | | Yes | |  |
| Clarke (2002)^47^ | Interventional | ENT | Yes | Structured communication tool / Clinical pathway | Development Implementation Description | 60 | | - | | - | | - | | - | | No | |  |
| Cohen (1997)^48^ | Interventional | head and neck | Yes | Clinical pathway | Implementation | 98 | |  | | - | | - | | - | | Yes | |  |
| Connolly et al (2009)^49^ | Observational | neurosurgery | No | Patient safety / Checklist | Development | 13 | | - | | - | | - | | - | | No | |  |
| Correa et al (1999)^50^ | Interventional | ENT | No | Clinical pathway | Development Implementation Description | 51 | |  | | - | | - | | - | | Yes | |  |
| Corsetti et al (1998)^51^ | Interventional | cardiac | No | Clinical pathway | Implementation Description | 851 | |  | | - | | - | | - | | No | |  |
| Cronin (1996)^52^ | Observational | thoracic | No | Clinical pathway | Description | - | | - | | - | | - | | - | | No | |  |
| Da Silva Fernades et al (2004)^53^ | Interventional | cardiac | No | Fast-track protocol | Implementation | 622 | |  | | - | | - | | - | | Yes | |  |
| D'Amato et al (1998)^54^ | Interventional | gynecology | No | Clinical pathway | Evaluation | 349 | |  | | - | |  | | - | | No | |  |
| Das-Neves-Pereira et al (2009)^55^ | Observational | thoracic | Yes | Fast-track protocol | Evaluation | 121 | |  | |  | |  | | - | | No | |  |
| de Vries et al (2010)^56^ | Interventional | multi-procedure | No | Clinical pathway / Checklst | Development Implementation Evaluation | 7580 | |  | |  | | - | |  | | No | |  |
| Delaney et al (2001)^57^ | Observational | colorectal | Yes | Fast-track protocol | Evaluation | 58 | |  | |  | |  | | - | | No | |  |
| Delaney et al (2003)^58^ | Randomized controlled trial | colorectal | No | Enhanced recover after surgery | Description Evaluation | 64 | |  | |  | |  | | - | | No | |  |
| Doerksen et al (2003)^59^ | Interventional | orthopedic | No | Clinical pathway | Development Implementation Evaluation | 47 | | - | | - | | - | | - | | No | |  |
| Douglas et al (2001)^60^ | Observational | orthopedic | No | Clinical pathway | Development Description | - | |  | | - | | - | | - | | No | |  |
| Dy et al (2003)^61^ | Interventional | multi-procedure | No | Clinical pathway | Evaluation | 10690 | |  | | - | | - | | - | | No | |  |
| Dy et al (2005)^62^ | Program Evaluation | multi-procedure | No | Clinical pathway | Comparison Evaluation | - | | - | | - | | - | | - | | No | |  |
| Eagle et al (1998)^63^ | Observational | cardiac | No | Clinical pathway | Implementation | 160 | |  | |  | | - | |  | | No | |  |
| Einav et al (2010)^64^ | Interventional | multi-procedure | No | Structured communication tool | Description Evaluation | 232 | | - | | - | | - | | - | | No | |  |
| Engelman et al (1994)^65^ | Interventional | cardiac | No | Fast-track protocol | Implementation | 562 | |  | | - | |  | |  | | No | |  |
| Ewing et al (2007)^66^ | Program Evaluation | multi-procedure | No | Clinical pathway | Development | - | | - | | - | | - | | - | | No | |  |
| Faiz et al (2009)^67^ | Observational | colorectal | Yes | Enhanced recover after surgery | Evaluation | 241 | |  | | - | | - | |  | | No | |  |
| Fearon (2005)^68^ | Qualitative | colorectal | No | Enhanced recover after surgery | Development Description | - | | - | | - | | - | | - | | No | |  |
| Feo et al (2009)^69^ | Interventional | colorectal | No | Fast-track protocol | Implementation | 100 | |  | |  | | - | |  | | No | |  |
| Ferri et al (2006)^70^ | Interventional | abdominal | No | Clinical pathway | Development Implementation Evaluation | | 49 | |  | |  | |  | | - | | Yes | |
| France et al (2008)^71^ | Program Evaluation | multi-procedure | No | Structured communication tool / Checklist | Evaluation | 30 | | - | | - | | - | | - | | No | |  |
| Frutos et al (2007)^72^ | Observational | bariatrics | No | Clinical pathway | Implementation Description | 311 | | - | | - | | - | | - | | No | |  |
| Gatt (2005)^73^ | Randomized controlled trial | colorectal | Yes | Enhanced recover after surgery | Evaluation | 39 | |  | |  | |  | |  | | No | |  |
| Goodwin et al (1999)^74^ | Program Evaluation | cardiac | No | Enhanced recover after surgery | Description | - | |  | | - | | - | | - | | No | |  |
| Gouvas et al (2009)^75^ | Systematic review | colorectal | Yes | Fast-track protocol | Evaluation | - | |  | |  | |  | |  | | No | |  |
| Graeber et al (2007)^76^ | Interventional | multi-procedure | Yes | Clinical pathway | Implementation | 129 | |  | | - | | - | | - | | Yes | |  |
| Gralla et al (2007)^77^ | Randomized controlled trial | urology | Yes | Fast-track protocol | Description | 50 | |  | | - | | - | | - | | No | |  |
| Guiahi et al (2010)^78^ | Interventional | gynecology | No | Patient care planning | Description Evaluation | 228 | | - | | - | | - | | - | | No | |  |
| Halm (1997)^79^ | Interventional | cardiac | No | Clinical pathway / Patient care planning | Development Implementation Evaluation | 159 | | - | |  | |  | |  | | No | |  |
| Hammer et al (2008)^80^ | Observational | colorectal | Yes | Fast-track protocol | Evaluation | 134 | |  | |  | | - | | - | | No | |  |
| Harvey (1990)^81^ | Program Evaluation | orthopedic | No | Clinical pathway | Implementation | - | | - | | - | | - | | - | | No | |  |
| Haynes et al (2009)^82^ | Interventional | multi-procedure | Yes | Checklist | Evaluation | 7688 | | - | |  | | - | |  | | No | |  |
| Healy (1998)^83^ | Interventional | orthopedic | No | Clinical pathway | Description Evaluation | 206 | |  | |  | | - | | - | | Yes | |  |
| Hedrick et al (2007)^84^ | Observational | abdominal | No | Patient safety | Development Implementation Description | 769 | |  | | - | | - | |  | | No | |  |
| Henderson et al (1999)^85^ | Observational | abdominal | No | Patient care planning | Development Description | 8 | | - | | - | | - | | - | | No | |  |
| Henrickson et al (2009)^86^ | Interventional | cardiac | No | Structured communication tool | Development Description | 16 | | - | | - | | - | | - | | No | |  |
| Holtzman (1998)^87^ | Interventional | abdominal | No | Clinical pathway | Description | 348 | |  | |  | | - | | - | | No | |  |
| Huber et al (1998)^88^ | Interventional | vascular | No | Clinical pathway | Development Implementation Description Evaluation | 65 | |  | |  | |  | |  | | Yes | |  |
| Husbands et al (1999)^89^ | Interventional | ENT | Yes | Clinical pathway | Implementation | 130 | |  | |  | |  | |  | | Yes | |  |
| Husted et al (2006)^90^ | Observational | orthopedic | No | Fast-track protocol | Implementation | 307 | |  | |  | |  | | - | | No | |  |
| Husted et al (2010)^91^ | Observational | orthopedic | No | Fast-track protocol | Evaluation | 1731 | |  | | - | |  | |  | | No | |  |
| Husted et al (2010)^92^ | Program Evaluation | orthopedic | No | Clinical pathway | Evaluation | - | | - | | - | | - | | - | | No | |  |
| Irizarry et al (1999)^93^ | Interventional | abdominal | No | Clinical pathway | Evaluation | 494 | |  | | - | |  | | - | | Yes | |  |
| Isozaki et al (1998)^94^ | Observational | cardiac | No | Clinical pathway | Development Description | - | | - | | - | | - | | - | | No | |  |
| Jakobsen et al (2006)^95^ | Interventional | colorectal | No | Fast-track protocol | Implementation Description | 160 | |  | | - | |  | | - | | No | |  |
| Jiang et al (2009)^96^ | Observational | abdominal | Yes | Fast-track protocol | Evaluation | 114 | |  | |  | | - | |  | | No | |  |
| Joh et al (2008)^97^ | Observational | colorectal | Yes | Clinical pathway | Implementation | 42 | | - | | - | | - | | - | | No | |  |
| Johnston et al (2009)^98^ | Observational | orthopedic | No | Preparatory pause | Evaluation | 231 | | - | | - | | - | | - | | No | |  |
| Jottard et al (2008)^99^ | Interventional | colorectal | No | Enhanced recover after surgery | Evaluation | 92 | |  | | - | | - | | - | | No | |  |
| Kallenbach et al (2000)^100^ | Observational | vascular | No | Clinical pathway | Development Description Evaluation | - | |  | | - | | - | | - | | Yes | |  |
| Kao et al (2010)^101^ | Interventional | multi-procedure | No | Clinical pathway / Checklist / Preparatory pause | Evaluation | 1052 | | - | | - | | - | | - | | No | |  |
| Kariv et al (2007)^102^ | Interventional |  | No | Fast-track protocol | Development Implementation Evaluation | 699 | |  | | - | |  | | - | | 0 | |  |
| Kawahara et al (2005)^103^ | Survey | colorectal | Yes | Clinical pathway | Evaluation | 52 | | - | | - | | - | | - | | No | |  |
| Kehlet et al (1999)^104^ | Observational | colorectal | Yes | Fast-track protocol | Evaluation | 16 | |  | | - | | - | | - | | No | |  |
| Kennedy et al (2007)^105^ | Interventional | abdominal | Yes | Clinical pathway | Implementation Description | 135 | |  | |  | |  | |  | | Yes | |  |
| Khoo et al (2007)^106^ | Randomized controlled trial | colorectal | Yes | Fast-track protocol | Implementation | 70 | |  | |  | |  | |  | | No | |  |
| Knight et al (2010)^107^ | Observational | multi-procedure | No | Patient safety | Evaluation | 112500 | | - | | - | | - | | - | | No | |  |
| Koval et al (2004)^108^ | Interventional | orthopedic | No | Clinical pathway | Description Evaluation | 1055 | |  | | - | | - | |  | | No | |  |
| Kremer et al (2005)^109^ | Observational | colorectal | No | Fast-track protocol | Implementation Description | 26 | |  | |  | | - | | - | | Yes | |  |
| Krezner (1999)^110^ | Observational | vascular | No | Clinical pathway | Development | - | |  | | - | |  | |  | | No | |  |
| Kulkarni et al (2011)^111^ | Interventional | endocrine | Yes | Clinical pathway | Evaluation | 681 | |  | |  | |  | | - | | Yes | |  |
| Larson et al (2010)^112^ | Interventional | colorectal | No | Fast-track protocol | Evaluation | 334 | |  | |  | |  | |  | | No | |  |
| Lazar et al (2001)^113^ | Interventional | cardiac | No | Fast-track protocol | Evaluation | 786 | |  | |  | |  | | - | | No | |  |
| Leibman et al (1998)^114^ | Interventional | prostate | Yes | Clinical pathway | Evaluation | 856 | |  | |  | |  | | - | | Yes | |  |
| Lemmens et al (2008)^115^ | Systematic review | colorectal | No | Clinical pathway | Evaluation | - | | - | | - | | - | | - | | Yes | |  |
| Lemmens et al (2009)^116^ | Systematic review | abdominal | Yes | Clinical pathway | Evaluation | - | |  | |  | |  | |  | | No | |  |
| Ley (1998)^117^ | Observational | cardiac | No | Fast-track protocol | Development Implementation Description Evaluation | - | |  | | - | | - | |  | | Yes | |  |
| Lindsetmo et al (2009)^118^ | Observational | colorectal | Yes | Clinical pathway | Evaluation | 37 | |  | | - | |  | |  | | No | |  |
| Lingard et al (2005)^119^ | Observational | vascular | No | Checklist | Development Implementation Description | - | | - | | - | | - | | - | | No | |  |
| Lingard et al (2006)^120^ | Observational | multi-procedure | No | Structured communication tool / Checklist | Implementation | - | | - | | - | | - | | - | | No | |  |
| Lingard et al (2008)^121^ | Interventional | multi-procedure | No | Structured communication tool / Checklist | Development | 172 | | - | | - | | - | | - | | No | |  |
| Liu et al (2010)^122^ | Interventional | abdominal | Yes | Enhanced recover after surgery | Implementation Description | 60 | |  | |  | |  | |  | | No | |  |
| Mabrey et al (1997)^123^ | Interventional | orthopedic | No | Clinical pathway | Implementation Description | 35 | |  | |  | |  | |  | | Yes | |  |
| MacKenzie et al (1995)^124^ | Program Evaluation | opthamology | Yes | Clinical pathway | Development Description | 139 | | - | | - | | - | | - | | No | |  |
| Maessen et al (2007)^125^ | Observational | colorectal | Yes | Enhanced recover after surgery | Implementation | 425 | |  | |  | |  | |  | | No | |  |
| Mandal et al (2004)^126^ | Interventional | opthamology | No | Patient care planning | Evaluation | 300 | | - | | - | |  | |  | | Yes | |  |
| Markey et al (2000)^127^ | Interventional | thyroid | No | Clinical pathway | Evaluation | 96 | |  | | - | |  | |  | | Yes | |  |
| Maruyama et al (2006)^128^ | Interventional | thoracic | Yes | Clinical pathway | Evaluation | 218 | |  | | - | | - | |  | | Yes | |  |
| Marx et al (2006)^129^ | Interventional | gynecology | Yes | Enhanced recover after surgery | Implementation Description | 141 | |  | |  | |  | |  | | No | |  |
| Marzen-Groller et al (2008)^130^ | Interventional | vascular | No | Clinical pathway | Implementation Description | 44 | |  | | - | | - | | - | | No | |  |
| Matsumoto et al (2002)^131^ | Interventional | vascular | No | Clinical pathway | Development Implementation Description Evaluation | 120 | |  | | - | | - | | - | | No | |  |
| Maxam- Moore et al (1996)^132^ | Program Evaluation | cardiac | No | Clinical pathway | Development Implementation Description | 32 | | - | | - | | - | | - | | No | |  |
| McAchran et al (2009)^133^ | Observational | urology | No | Fast-track protocol | Evaluation | 86 | |  | | - | |  | | - | | No | |  |
| McLellan et al (2006)^134^ | Interventional | urology | Yes | Clinical pathway | Evaluation | 215 | |  | |  | |  | | - | | No | |  |
| Melbert et al (2002)^135^ | Interventional | colorectal | No | Clinical pathway | Development Implementation Description Evaluation | 385 | |  | |  | |  | |  | | Yes | |  |
| Mikulaninec (1992)^136^ | Observational | vascular | No | Clinical pathway | Description | 21 | |  | | - | | - | | - | | No | |  |
| Mo et al (2010)^137^ | Interventional | thoracic | No | Clinical pathway | Development Implementation Description | 60 | | - | |  | | - | | - | | No | |  |
| Muehling et al (2008)^138^ | Randomized controlled trial | thoracic | Yes | Fast-track protocol | Implementation | 58 | |  | |  | | - | |  | | No | |  |
| Muehling et al (2008)^139^ | Randomized controlled trial | vascular | No | Fast-track protocol | Implementation | 79 | |  | |  | | - | |  | | No | |  |
| Muehling et al (2009)^140^ | Randomized controlled trial | vascular | No | Fast-track protocol | Implementation | 96 | |  | |  | |  | |  | | No | |  |
| Muller et al (2009)^141^ | Randomized controlled trial | colorectal | No | Fast-track protocol | Development Implementation Evaluation | 151 | |  | | - | |  | | - | | No | |  |
| Munitiz et al (2010)^142^ | Interventional | abdominal | Yes | Clinical pathway | Implementation Description | 148 | |  | |  | |  | |  | | No | |  |
| Murphy et al (2007)^143^ | Interventional | vascular | No | Fast-track protocol | Implementation Description | 30 | |  | | - | |  | | - | | No | |  |
| Musser et al (1996)^144^ | Interventional | vascular | No | Clinical pathway | Description Evaluation | 102 | |  | |  | |  | |  | | Yes | |  |
| Nagpal et al (2010)^145^ | Systematic review | multi-procedure | No | Structured communication tool | Evaluation | - | | - | | - | | - | | - | | No | |  |
| Naughton et al (2005)^146^ | Observational | cardiac | No | Enhanced recover after surgery | Description Evaluation | 100 | |  | |  | | - | | - | | No | |  |
| Neily et al (2010)^147^ | Program Evaluation | multi-procedure | No | Structured communication tool | Development Implementation Evaluation | 108 | | - | | - | | - | | - | | No | |  |
| Nilsson et al (2010)^148^ | Program Evaluation | multi-procedure | No | Preparatory pause | Evaluation | 331 | | - | | - | | - | | - | | No | |  |
| Nuelle et al (2007)^149^ | Interventional | orthopedic | No | Patient care planning | Implementation Description Evaluation | 50 | |  | | - | | - | | - | | No | |  |
| Nygren et al (2005)^150^ | Program Evaluation | colorectal | Yes | Enhanced recover after surgery | Comparison | 451 | |  | |  | |  | |  | | No | |  |
| Okita et al (2009)^151^ | Observational | thoracic | Yes | Clinical pathway | Evaluation | 61 | | - | | - | | - | |  | | No | |  |
| Oldmeadow et al (2004)^152^ | Interventional | orthopedic | No | Clinical pathway | Description | 100 | |  | | - | | - | | - | | No | |  |
| Ottesen et al (2002)^153^ | Observational | gynecology | No | Clinical pathway | Implementation | 41 | |  | | - | | - | | - | | No | |  |
| Paige et al (2008)^154^ | Interventional | multi-procedure | No | Structured communication tool | Description Evaluation | 36 | | - | | - | | - | | - | | No | |  |
| Paige et al (2009)^155^ | Interventional | multi-procedure | No | Structured communication tool / Preparatory pause | Evaluation | 36 | | - | | - | | - | | - | | No | |  |
| Paone et al (1998)^156^ | Program Evaluation | vascular | No | Clinical pathway | Evaluation | 445 | | - | | - | | - | | - | | No | |  |
| Paull et al (2010)^157^ | Observational | multi-procedure | No | Structured communication tool / Checklist | Evaluation | - | | - | | - | | - | | - | | No | |  |
| Pearson et al (2000)^158^ | Interventional | orthopedic | No | Clinical pathway | Development Evaluation | 177 | |  | |  | |  | | - | | No | |  |
| Pearson et al (2001)^159^ | Interventional | multi-procedure | No | Clinical pathway | Development Implementation Description Comparison Evaluation | 6796 | |  | | - | |  | |  | | Yes | |  |
| Pedersen et al (2008)^160^ | Interventional | orthopedic | No | Clinical pathway / Patient care planning | Description Evaluation | 535 | |  | | - | | - | |  | | No | |  |
| Petersen et al (2008)^161^ | Interventional | orthopedic | No | Clinical pathway | Implementation | 61 | | - | | - | | - | | - | | No | |  |
| Pitt et al (1999)^162^ | Interventional | abdominal | Yes | Clinical pathway | Implementation | 339 | |  | | - | | - | |  | | Yes | |  |
| Podore et al (1999)^163^ | Observational | vascular | No | Clinical pathway | Description | 50 | |  | | - | |  | | - | | No | |  |
| Polle et al (2007)^164^ | Interventional | colorectal | Yes | Fast-track protocol | Implementation Description | 107 | |  | |  | |  | |  | | No | |  |
| Porter et al (2000)^165^ | Interventional | abdominal | Yes | Clinical pathway | Evaluation | 148 | |  | |  | |  | |  | | Yes | |  |
| Pritts et al (1999)^166^ | Interventional | colorectal | No | Clinical pathway | Implementation | 268 | |  | |  | |  | |  | | Yes | |  |
| Prouty et al (2006)^167^ | Observational | orthopedic | No | Structured communication tool | Implementation | 944 | | - | | - | | - | | - | | No | |  |
| Pruthi et al (2003)^168^ | Interventional | urology | Yes | Clinical pathway | Implementation Description | 40 | |  | | - | | - | | - | | No | |  |
| Pruthi et al (2010)^169^ | Observational | urology | No | Enhanced recover after surgery | Implementation | 362 | |  | |  | | - | |  | | No | |  |
| Quigley et al (1997)^170^ | Interventional | cardiac | No | Fast-track protocol | Evaluation | 532 | |  | | - | |  | |  | | Yes | |  |
| Raue et al (2004)^171^ | Interventional | colorectal | No | Fast-track protocol | Description Evaluation | 52 | |  | |  | |  | |  | | No | |  |
| Recart et al (2005)^172^ | Randomized controlled trial | urology | Yes | Fast-track protocol | Implementation | 25 | |  | | - | |  | | - | | No | |  |
| Renkawitz et al (2010)^173^ | Interventional | orthopedic | No | Checklist / Enhanced recover after surgery | Description Comparison | 143 | |  | | - | |  | | - | | No | |  |
| Riddle et al (1996)^174^ | Interventional | cardiac | No | Enhanced recover after surgery | Development Implementation Evaluation | 615 | |  | | - | | - | | - | | Yes | |  |
| Rogers et al (1998)^175^ | Observational | cardiac | No | Clinical pathway | Description Evaluation | 69 | | - | | - | | - | | - | | No | |  |
| Rogers et al (2000)^176^ | Interventional | ENT | No | Clinical pathway | Description Evaluation | 955 | | - | | - | | - | | - | | No | |  |
| Ronellenfitsch et al (2008)^177^ | Systematic review | multi-procedure | No | Clinical pathway | Evaluation | - | | - | | - | | - | | - | | Yes | |  |
| Rouse et al (1998)^178^ | Interventional | bariatrics | No | Clinical pathway | Implementation | 32 | |  | | - | |  | | - | | Yes | |  |
| Rusynko et al (2004)^179^ | Program Evaluation | multi-procedure | No | Preparatory pause | Development Description | - | | - | | - | | - | | - | | No | |  |
| Sakallaris et al (2000)^180^ | Interventional | cardiac | No | Clinical pathway | Description Evaluation | 1297 | |  | | - | | - | | - | | Yes | |  |
| Santoso et al (2002)^181^ | Interventional | breast | Yes | Clinical pathway | Development Implementation Description Evaluation | 152 | |  | |  | |  | | - | | Yes | |  |
| Scatizzi et al (2009)^182^ | Observational | colorectal | No | Fast-track protocol | Implementation Description | 101 | |  | |  | | - | |  | | No | |  |
| Scharfenberg et al (2007)^183^ | Observational | colorectal | Yes | Fast-track protocol | Implementation | 74 | |  | |  | | - | | - | | No | |  |
| Schneider et al (2009)^184^ | Observational | orthopedic | No | Clinical pathway | Evaluation | 100 | |  | |  | |  | | - | | No | |  |
| Schwarzbach et al (2010)^185^ | Interventional | abdominal | No | Clinical pathway | Implementation Description | 76 | |  | |  | | - | |  | | No | |  |
| Schwenk et al (2006)^186^ | Observational | colorectal | Yes | Fast-track protocol | Implementation Description | 70 | |  | |  | |  | | - | | No | |  |
| Schwenk et al (2008)^187^ | Program Evaluation | colorectal | Yes | Enhanced recover after surgery | Description Evaluation | 1047 | | - | |  | | - | | - | | No | |  |
| Serclova et al (2009)^188^ | Randomized controlled trial | colorectal | Yes | Enhanced recover after surgery | Evaluation | 103 | |  | |  | |  | |  | | No | |  |
| Sherman et al (2001)^189^ | Interventional | ENT | Yes | Clinical pathway | Development Evaluation | 62 | |  | |  | |  | |  | | No | |  |
| Sladek et al (1999)^190^ | Observational | endocrine | Yes | Clinical pathway / Patient care planning | Implementation Description Evaluation | 11 | | - | | - | | - | | - | | No | |  |
| So et al (2008)^191^ | Interventional | abdominal | Yes | Clinical pathway | Implementation | 115 | |  | |  | |  | |  | | Yes | |  |
| Soria et al (2005)^192^ | Interventional | abdominal | Yes | Clinical pathway | Description Evaluation | 300 | |  | |  | |  | |  | | Yes | |  |
| Spanjersberg et al (2011)^193^ | Systematic review | colorectal | Yes | Enhanced recover after surgery | Comparison | 237 | |  | |  | |  | |  | | No | |  |
| Stanford et al (2009)^194^ | Interventional | cardiac | No | Checklist | Evaluation | 1085 | | - | | - | | - | |  | | No | |  |
| Stephen et al (2003)^195^ | Interventional | colorectal | Yes | Clinical pathway | Development Implementation Evaluation | 138 | |  | |  | |  | |  | | Yes | |  |
| Stidham et al (2001)^196^ | Observational | neurosurgery | No | Clinical pathway | Description Evaluation | 59 | |  | |  | |  | |  | | No | |  |
| Strong et al (1991)^197^ | Observational | cardiac | No | Clinical pathway | Evaluation | 28 | |  | | - | | - | | - | | No | |  |
| Tan et al (2008)^198^ | Interventional | colorectal | No | Clinical pathway | Implementation | 408 | |  | | - | |  | |  | | Yes | |  |
| Teeuwen et al (2010)^199^ | Observational | colorectal | Yes | Enhanced recover after surgery | Evaluation | 183 | |  | |  | |  | |  | | No | |  |
| Thomas (2003)^200^ | Observational | orthopedic | No | Clinical pathway | Evaluation | 215 | |  | | - | | - | | - | | No | |  |
| Tomaszek et al (2010)^201^ | Interventional | ENT | Yes | Clinical pathway | Description Comparison | 386 | |  | |  | |  | | - | | No | |  |
| Topal et al (2007)^202^ | Interventional | abdominal | No | Clinical pathway | Implementation | 811 | |  | |  | |  | |  | | Yes | |  |
| Toraman et al (2005)^203^ | Observational | cardiac | No | Fast track protocol | Implementation | 299 | | - | | - | | - | | - | | No | |  |
| Tromp et al (2003)^204^ | Program Evaluation | cardiac | No | Structured communication tool / Patient care planning | Development Implementation Description | 61 | | - | | - | | - | | - | | No | |  |
| Trussel et al (2008)^205^ | Interventional | cardiac | No | Enhanced recover after surgery | Implementation | 1482 | | - | | - | | - | | - | | No | |  |
| Uchiyama et al (2002)^206^ | Interventional | abdominal | No | Clinical pathway | Evaluation | 369 | |  | | - | | - | | - | | Yes | |  |
| Ueda et al (2003)^207^ | Observational | thoracic | Yes | Clinical pathway | Evaluation | 40 | | - | | - | | - | | - | | No | |  |
| Van Dam et al (2008)^208^ | Interventional | abdominal | Yes | Enhanced recover after surgery | Description Evaluation | 161 | |  | |  | |  | |  | | No | |  |
| Van Herck et al (2010)^209^ | Systematic review | orthopedic | No | Clinical pathway | Description Comparison Evaluation | - | |  | | - | | - | | - | | Yes | |  |
| Varadhan et al (2010)^210^ | Program Evaluation | colorectal | No | Enhanced recover after surgery | Description | 452 | |  | |  | |  | |  | | No | |  |
| Varadhan et al (2010)^211^ | Systematic review | colorectal | No | Enhanced recover after surgery | Evaluation | 452 | |  | |  | |  | |  | | No | |  |
| Veltman et al (1999)^212^ | Observational | abdominal | No | Clinical pathway | Development Evaluation | 60 | |  | |  | | - | | - | | Yes | |  |
| Wadhera et al (2010)^213^ | Interventional | cardiac | No | Structured communication tool | Implementation | 34 | | - | | - | | - | | - | | No | |  |
| Wainwright et al (2010)^214^ | Observational | orthopedic | No | Enhanced recover after surgery | Evaluation | 2391 | |  | |  | |  | | - | | No | |  |
| Walsh et al (2001)^215^ | Interventional | vascular | No | Clinical pathway | Development Comparison | 194 | |  | | - | |  | | - | | No | |  |
| Walter et al (2006)^216^ | Interventional | orthopedic | No | Clinical pathway | Implementation | 1680 | |  | | - | | - | | - | | No | |  |
| Wang et al (2010)^217^ | Randomized controlled trial | abdominal | Yes | Enhanced recover after surgery | Implementation Description | 92 | |  | |  | |  | |  | | Yes | |  |
| Watters et al (2006)^218^ | Observational | orthopedic | No | Structured communication tool / Clinical pathway / Patient care planning | Development Implementation Description | - | |  | | - | | - | | - | | Yes | |  |
| Webster et al (2005)^219^ | Observational | urology | No | Clinical pathway | Evaluation | 39 | |  | | - | |  | | - | | No | |  |
| Wehberg et al (2009)^220^ | Interventional | cardiac | No | Fast track protocol | Implementation | 45 | |  | |  | |  | |  | | Yes | |  |
| Weiser et al (2010)^221^ | Interventional | multi-procedure | No | Checklist | Implementation Description | 1750 | | - | | - | | - | |  | | No | |  |
| White et al (2009)^222^ | Qualitative | opthamology | No | Patient safety | Evaluation | 69 | | - | | - | | - | | - | | No | |  |
| Wichmann et al (2006)^223^ | Interventional | abdominal | Yes | Enhanced recover after surgery | Description Evaluation | 24 | |  | | - | | - | | - | | No | |  |
| Wichmann et al (2007)^224^ | Interventional | colorectal | Yes | Enhanced recover after surgery | Evaluation | 40 | |  | |  | | - | | - | | No | |  |
| Wind et al (2006)^225^ | Systematic review | colorectal | Yes | Enhanced recover after surgery | Evaluation | - | |  | |  | |  | |  | | No | |  |
| Wright et al (1997)^226^ | Interventional | thoracic | No | Clinical pathway | Development Implementation | 277 | |  | |  | |  | |  | | Yes | |  |
| Yanagi et al (2007)^227^ | Interventional | abdominal | No | Clinical pathway | Evaluation | 145 | |  | | - | | - | | - | | No | |  |
| Yanatori et al (2007)^228^ | Interventional | cardiac | No | Fast-track protocol | Implementation Description | 94 | |  | |  | |  | |  | | Yes | |  |
| Yang et al (2007)^229^ | Interventional | orthopedic | No | Patient safety | Evaluation | 794 | | - | | - | | - | | - | | Yes | |  |
| Zargar-Shoshtari et al (2008)^230^ | Observational | colorectal | Yes | Fast-track protocol | Implementation Description | 30 | |  | | - | | - | | - | | No | |  |
| Zargar-Shoshtari et al (2009)^231^ | Interventional | colorectal | No | Enhanced recover after surgery | Implementation | 52 | |  | | - | |  | |  | | No | |  |
| Zargar-Shoshtori et al (2008)^232^ | Interventional | colorectal | No | Enhanced recover after surgery | Description Evaluation | 100 | |  | |  | |  | |  | | No | |  |

**REFERENCES**

1. Aboulian A, Hassan Z, Lin MY, Kaji AH, Kumar RR, Aboulian A, Hassan Z, Lin MYC, Kaji AH, Kumar RR. Successful enhanced recovery program after colorectal surgery in a county institution. *Am Surg* 2010;**76**(10): 1158-1162.

2. Ad N, Henry L, Hunt S, Stone L. The implementation of a comprehensive clinical protocol improves long-term success after surgical treatment of atrial fibrillation. *J Thorac Cardiovasc Surg* 1146;**139**(5): 1146-1152.

3. Aguilar-Nascimento JE, Salomao AB, Caporossi C, Diniz BN. Clinical benefits after the implementation of a multimodal perioperative protocol in elderly patients. *Arquivos de Gastroenterologia* 2010;**47**(2): 178-183.

4. Al Chalabi H, Kavanagh DO, Hassan L, Donnell KO, Nugent E, Andrews E, Keane FBV, O'Riordain DS, Miller A, Neary P. The benefit of an enhanced recovery programme following elective laparoscopic sigmoid colectomy. *Int J Colorectal Dis* 2010;**25** (6): 761-766.

5. Altpeter T, Luckhardt K, Lewis JN, Harken AH, Polk Jr HC. Expanded Surgical Time Out: A Key to Real-Time Data Collection and Quality Improvement. *J Am Coll Surg* 2007;**204 (4)**: 527-532.

6. Andersen J, Kehlet H. Fast track open ileo-colic resections for Crohn's disease. *Colorectal Disease* 2005(of Publication: Jul 2005): 7 (4) (pp 394-397), 2005.

7. Anderson A, McNaught, CE., MacFie, J., Tring, I., Barker, P., Mitchell, CJ. Randomized clinical trial of multimodal optimization and standard perioperative surgical care. *Brit J Surg* 2003;**90**(12): 1497-1504.

8. Archer S. Implementation of a clinical pathway decreases length of stay and hospital charges for patients undergoing total colectomy and ileal pouch/anal anastomosis. *Surgery* 1997;**122**: 699-703.

9. Austin Health Post-Operative Surveillance Team (POST) Investigators. Methodology for a study of structured co-management of high-risk postoperative patients in a teaching hospital. [Review]. *Critical Care & Resuscitation* 2010;**12**(4): 277-286.

10. Back MR, Harward TR, Huber TS, Carlton LM, Flynn TC, Seeger JM. Improving the cost-effectiveness of carotid endarterectomy. *J Vasc Surg* 1997;**26**(3): 456-462.

11. Backster A, Teo A, Swift M, Polk HC, Jr., Harken AH, Backster A, Teo A, Swift M, Polk HC, Jr., Harken AH. Transforming the surgical "time-out" into a comprehensive "preparatory pause". *J Card Surg* 2007;**22**(5): 410-416.

12. Baird G, Maxson P, Wrobleski D, Luna BS. Fast-track colorectal surgery program reduces hospital length of stay. *Clinical Nurse Specialist* 2010;**24**(4): 202-208.

13. Baker B, Fillion B, Davitt K, Finnestad L. Ambulatory surgical clinical pathway. *J Perianesth Nurs* 1999;**14** (1): 2-11.

14. Balzano G, Zerbi A, Braga M, Rocchetti S, Beneduce AA, Di Carlo V. Fast-track recovery programme after pancreatico- duodenectomy reduces delayed gastric emptying. *British Journal of Surgery* 2008;**95**(11): 1387-1393.

15. Basse L, Hjort Jakobsen D, Billesbolle P, Werner M, Kehlet H. A clinical pathway to accelerate recovery after colonic resection. *Ann Surg* 2000;**232**(1): 51-57.

16. Basse L. Accelerated postoperative recovery programme after colonic resection improves physical performance, pulmonary function and body composition. . *Br J Surg* 2002;**89**: 446–453.

17. Basse L, Jacobsen DH, Billesbolle P, Kehlet H. Colostomy closure after Hartmann's procedure with fast-track rehabilitation. *Diseases of the Colon and Rectum* 2002(of Publication: 01 Dec 2002): 45 (12) (pp 1661-1664), 2002.

18. Basse L, Thorbol JE, Lossl K, Kehlet H. Colonic surgery with accelerated rehabilitation or conventional care.[Erratum appears in Dis Colon Rectum. 2005 Aug;48(8):1673].[Erratum appears in Dis Colon Rectum. 2004 Jun;47(6):951]. *Diseases of the Colon & Rectum* 2004;**47**(3): 271-277.

19. Beaupre LA, Cinats JG, Senthilselvan A, Lier D, Jones CA, Scharfenberger A, Johnston DW, Saunders LD. Reduced morbidity for elderly patients with a hip fracture after implementation of a perioperative evidence-based clinical pathway. *Qual Saf Health Care* 2006;**15**(5): 375-379.

20. Becker BN, Breiterman-White R, Nylander W, Van Buren D, Fotiadis C, Richie RE, Schulman G. Care pathway reduces hospitalizations and cost for hemodialysis vascular access surgery. *Am J Kidney Dis* 1997;**30**(4): 525-531.

21. Berger RA, Sanders S, D'Ambrogio E, Buchheit K, Deirmengian C, Paprosky W, Della Valle CJ, Rosenberg AG. Minimally invasive quadriceps-sparing TKA: results of a comprehensive pathway for outpatient TKA. *J Knee Surg* 2006;**19**(2): 145-148.

22. Berger RA, Sanders SA, Thill ES, Sporer SM, Della Valle C. Newer anesthesia and rehabilitation protocols enable outpatient hip replacement in selected patients. *Clin Orthop Relat Res* 2009;**467 (6)**: 1424-1430.

23. Berger RA, Kusuma SK, Sanders SA, Thill ES, Sporer SM. The feasibility and perioperative complications of outpatient knee arthroplasty. *Clin Ortho and Relat Res* 2009;**467 (6)**: 1443-1449.

24. Berry SA, Doll MC, McKinley KE, Casale AS, Bothe A, Jr. ProvenCare: quality improvement model for designing highly reliable care in cardiac surgery. *Qual Safety Health Care* 2009;**18**(5): 360-368.

25. Bleakley A, Boyden J, Hobbs A, Walsh L, Allard J, Bleakley A, Boyden J, Hobbs A, Walsh L, Allard J. Improving teamwork climate in operating theatres: the shift from multiprofessionalismto interprofessionalism. *J Interprof Care* 2006;**20**(5): 461-470.

26. Braumann C, Guenther N, Wendling P, Engemann R, Germer CT, Probst W, Mayer HP, Rehnisch B, Schmid M, Nagel K, Schwenk W, Fast-Track Colon IIQAG. Multimodal perioperative rehabilitation in elective conventional resection of colonic cancer: results from the German Multicenter Quality Assurance Program 'Fast-Track Colon II'. *Dig Surg* 2009;**26**(2): 123-129.

27. Broder MS, Bovone S. Improving treatment outcomes with a clinical pathway for hysterectomy and myomectomy. *J Reprod Med* 1003;**47**(12): 999-1003.

28. Brown B, Riippa M, Shaneberger K. Promoting patient safety through preoperative patient verification. *AORN Journal* 2001;**74** (5): 690-698.

29. Brustia P, Renghi A, Fassiola A, Gramaglia L, Della Corte F, Cassatella R, Cumino A. Fast-track approach in abdominal aortic surgery: left subcostal incision with blended anesthesia. *Interactive Cardiovascular & Thoracic Surgery* 2007;**6**(1): 60-64.

30. Bryan S, Holmes S, Postlethwaite D, Carty N. A breast unit care pathway: enhancing the role of the nurse. *Prof Nurse* 2002;**18 (3)**: 151-154.

31. Buzink SN, van Lier L, de Hingh IH, Jakimowicz JJ, Buzink SN, van Lier L, de Hingh IHJT, Jakimowicz JJ. Risk-sensitive events during laparoscopic cholecystectomy: the influence of the integrated operating room and a preoperative checklist tool. *Surg Endosc* 2010;**24**(8): 1990-1995.

32. Cabello CC, Tahan HA. Implementation of an interdisciplinary clinical pathway for patients after a liver transplant. *Nurs Case Manag* 1998;**3**(6): 255-265.

33. Calland JF, Tanaka K, Foley E, Bovbjerg VE, Markey DW, Blome S, Minasi JS, Hanks JB, Moore MM, Young JS, Jones RS, Schirmer BD, Adams RB. Outpatient laparoscopic cholecystectomy: patient outcomes after implementation of a clinical pathway. *Ann Surg* 2001;**233**(5): 704-715.

34. Calligaro KD, Dougherty MJ, Raviola CA, Musser DJ, DeLaurentis DA. Impact of clinical pathways on hospital costs and early outcome after major vascular surgery. *J Vas Surg* 1995;**22**(6): 649-657.

35. Calligaro KD, Doerr KJ, McAfee-Bennett S, Mueller K, Dougherty MJ. Critical pathways can improve results with carotid endarterectomy. *Semin Vasc Surg* 2004;**17**(3): 253-256.

36. Carli F, Charlebois P, Baldini G, Cachero O, Stein B. An integrated multidisciplinary approach to implementation of a fast-track program for laparoscopic colorectal surgery. *Canadian Journal of Anaesthesia* 2009;**56**(11): 837-842.

37. Carter J, Szabo R, Sim WW, Pather S, Philp S, Nattress K, Cotterell S, Patel P, Dalrymple C. Fast track surgery: a clinical audit. *Australian & New Zealand Journal of Obstetrics & Gynaecology* 2010;**50**(2): 159-163.

38. Cayir G, Beji NK, Yalcin O, Cayir G, Beji NK, Yalcin O. Effectiveness of nursing care after surgery for stress urinary incontinence. *Urol Nurs* 2007;**27**(1): 25-33.

39. Cerfolio RJ, Pickens A, Bass C, Katholi C, Vallieres E, Rice T, Brantigan C. Fast-tracking pulmonary resections. *Journal of Thoracic and Cardiovascular Surgery* 2001(of Publication: 01 Aug 2001): 122 (122) (pp 318-324), 2001.

40. Cerfolio RJ, Bryant AS, Bass CS, Alexander JR, Bartolucci AA. Fast tracking after Ivor Lewis esophagogastrectomy. *Chest* 2004;**126**(4): 1187-1194.

41. Chalian AA, Kagan SH, Goldberg AN, Gottschalk A, Dakunchak A, Weinstein GS, Weber RS, Chalian AA, Kagan SH, Goldberg AN, Gottschalk A, Dakunchak A, Weinstein GS, Weber RS. Design and impact of intraoperative pathways for head and neck resection and reconstruction. *Arch Otolaryngol Head Neck Surg* 2002;**128**(8): 892-896.

42. Chang PL, Wang TM, Huang ST, Hsieh MLI, Tsui KH, Lai RH. Effects of implementation of 18 clinical pathways on costs and quality of care among patients undergoing urological surgery. *J Urol* 1999;**161** (6): 1858-1862.

43. Chang WC, Lin CC. A clinical pathway for laparoscopically assisted vaginal hysterectomy. Impact on costs and clinical outcome. *J Reprod Med* 2003;**48**(4): 247-251.

44. Chen AY, Callender D, Mansyur C, Reyna KM, Limitone E, Goepfert H. The impact of clinical pathways on the practice of head and neck oncologic surgery: The University of Texas M.D. Anderson Cancer Center experience. *Arch Otolaryngol Head and Neck Surg* 2000;**126** (3): 322-326.

45. Claridge JA, Young JS. A successful multimodality strategy for management of liver injuries. *Am Surg* 2000;**66**(10): 920-925.

46. Clark JA, Kotyra LG, Brocious T. Rapid progression following cardiac surgery. *Critical Care Nursing Clinics of North America* 1999;**11**(2): 159-175.

47. Clarke LK. Pathways for head and neck surgery: a patient-education tool. *Clin J Oncol Nurs* 2002;**6 (2)**: 78-82.

48. Cohen J. Critical pathways for head and neck surgery: development and implementation *Arch Otolaryngol Head Neck Surg* 1997;**123**: 11-14.

49. Connolly PJ, Kilpatrick M, Jaggi JL, Church E, Baltuch GH. Feasibility of an operational standardized checklist for movement disorder surgery: A pilot study. *Stereotact Funct Neurosurg* 2009;**87 (2)**: 94-100.

50. Correa AJ, Reinisch L, Paty VA, Sanders DL, Duncavage JA. Analysis of a critical pathway in osteoplastic flap for frontal sinus obliteration. *Laryngoscope* 1999;**109**(8): 1212-1216.

51. Corsetti AL, Perry D. A comprehensive approach to facilitating the recovery of cardiac surgery patients. *J Cardiovasc Nurs* 1998;**12**(3): 82-90.

52. Cronin AL. Lung volume reduction surgery CareMap. A multidisciplinary approach to managing patient care. *Critical Care Nursing Clinics of North America* 1996;**8**(3): 333-343.

53. Da Silva Fernandes AM, Mansur AJ, Caneo LF, Dias Lourenco D, Albuquerque Piccioni M, Franchi SM, Afiune CMC, Gadioli JW, De Almeida Oliveira S, Franchini Ramires JA. The reduction in hospital stay and costs in the care of patients with congenital heart diseases undergoing fast-track cardiac surgery. [Portuguese, English]. *Arquivos Brasileiros de Cardiologia* 2004(of Publication: Jul 2004): 83 (81) (pp 18-34), 2004.

54. D'Amato LO, Jr., Talmage LA, Hyde K, McKnight S, Vandenbusche P. Outcomes in abdominal hysterectomy patients with benign disease. Use of physician-developed clinical protocols. *Journal of Reproductive Medicine* 1998;**43**(11): 975-985.

55. Das-Neves-Pereira JC, Bagan P, Coimbra-Israel AP, Grimaillof-Junior A, Cesar-Lopez G, Milanez-de-Campos JR, Riquet M, Biscegli-Jatene F. Fast-track rehabilitation for lung cancer lobectomy: a five-year experience. *European Journal of Cardio Thoracic Surgery* 2009;**36**(2): 383-391.

56. de Vries EN, Prins HA, M.P.H. CR, J. dOA, van Andel G, van Helden SH, Schlack WS, van Putten MA, Gouma DJ, Dijkgraaf MG, Smorenburg SM, Boermeester MA. Effect of a Comprehensive Surgical Safety System on Patient Outcomes. *N Engl J Med* 2010;**363**(20): 1928-1937.

57. Delaney CP, Fazio VW, Senagore AJ, Robinson B, Halverson AL, Remzi FH. 'Fast track' postoperative management protocol for patients with high co-morbidity undergoing complex abdominal and pelvic colorectal surgery. *British Journal of Surgery* 2001(of Publication: 2001): 88 (11) (pp 1533-1538), 2001.

58. Delaney CP, Zutshi M, Senagore AJ, Remzi FH, Hammel J, Fazio VW. Prospective, randomized, controlled trial between a pathway of controlled rehabilitation with early ambulation and diet and traditional postoperative care after laparotomy and intestinal resection. *Dis Colon Rectum* 2003;**46**(7): 851-859.

59. Doerksen K, Dusik-Sharpe J. Lumbar discectomy: developing and implementing a day surgery protocol. *Axon* 2003;**25**(1): 18-21.

60. Douglas P, Asimus M, Swan J, Spigelman A. Prevention of orthopaedic wound infections: a quality improvement project. *J Qual Clin Pract* 2001;**21**(4): 149-153.

61. Dy SM, Garg PP, Nyberg D, Dawson PB, Pronovost PJ, Morlock L, Rubin HR, Diener-West M, Wu AW. Are critical pathways effective for reducing postoperative length of stay? *Med Care* 2003;**41**(5): 637-648.

62. Dy SM, Garg P, Nyberg D, Dawson PB, Pronovost PJ, Morlock L, Rubin H, Wu AW, Dy SM, Garg P, Nyberg D, Dawson PB, Pronovost PJ, Morlock L, Rubin H, Wu AW. Critical pathway effectiveness: assessing the impact of patient, hospital care, and pathway characteristics using qualitative comparative analysis. *Health Serv Res* 2005;**40**(2): 499-516.

63. Eagle KA, Moscucci M, Kline-Rogers E, Chaffee BW, Barry PA, Roberts S, Froehlich J, Cornish LA, Wurster H, Deeb GM. Evaluating and improving the delivery of heart care: the University of Michigan experience. *American Journal of Managed Care* 1998;**4**(9): 1300-1309.

64. Einav Y, Gopher D, Kara I, Ben-Yosef O, Lawn M, Laufer N, Liebergall M, Donchin Y, Einav Y, Gopher D, Kara I, Ben-Yosef O, Lawn M, Laufer N, Liebergall M, Donchin Y. Preoperative briefing in the operating room: shared cognition, teamwork, and patient safety. *Chest* 2010;**137**(2): 443-449.

65. Engelman RM, Rousou JA, Flack JE, 3rd, Deaton DW, Humphrey CB, Ellison LH, Allmendinger PD, Owen SG, Pekow PS. Fast-track recovery of the coronary bypass patient. *Annals of Thoracic Surgery* 1994;**58**(6): 1742-1746.

66. Ewing H, Bruder G, Baroco P, Hill M, Sparkman LP, Ewing H, Bruder G, Baroco P, Hill M, Sparkman LP. Eliminating perioperative adverse events at Ascension Health. *Jt Comm J Qual Patient Saf* 2007;**33**(5): 256-266.

67. Faiz O, Brown T, Colucci G, Kennedy RH. A cohort study of results following elective colonic and rectal resection within an enhanced recovery programme. *Colorectal Disease* 2009(of Publication: 2009): 11 (14) (pp 366-372), 2009.

68. Fearon K. Enhanced recovery after surgery: a consensus review of clinical care for patients undergoing colonic resection. . *Clin Nutr* 2005;**24**: 466–477.

69. Feo CV, Lanzara S, Sortini D, Ragazzi R, De Pinto M, Pansini GC, Liboni A. Fast track postoperative management after elective colorectal surgery: a controlled trail. *Am Surg* 2009;**75**(12): 1247-1251.

70. Ferri LE, Feldman LS, Stanbridge DD, Fried GM. Patient perception of a clinical pathway for laparoscopic foregut surgery. *J Gastrointest Surg* 2006;**10**(6): 878-882.

71. France DJ, Leming-Lee S, Jackson T, Feistritzer NR, Higgins MS, France DJ, Leming-Lee S, Jackson T, Feistritzer NR, Higgins MS. An observational analysis of surgical team compliance with perioperative safety practices after crew resource management training. *Am J Surg* 2008;**195**(4): 546-553.

72. Frutos MD, Lujan J, Hernandez Q, Valero G, Parrilla P. Clinical pathway for laparoscopic gastric bypass. *Obes Surg* 2007;**17**(12): 1584-1587.

73. Gatt M. Randomized clinical trial of multimodal optimization of surgical care inpatients undergoing major colonic resection. *Br J Surg* 2005;**952**: 1354-1362.

74. Goodwin MJ, Bissett L, Mason P, Kates R, Weber J. Early extubation and early activity after open heart surgery. *Critical Care Nurse* 1999;**19**(5): 18-26.

75. Gouvas N, Tan E, Windsor A, Xynos E, Tekkis PP. Fast-track vs standard care in colorectal surgery: A meta-analysis update. *International Journal of Colorectal Disease* 2009(of Publication: 2009): 24 (10) (pp 1119-1131), 2009.

76. Graeber S, Richter S, Folz J, Pham PT, Jacob P, Schilling MK. Clinical pathways in general surgery: Development, implementation, and evaluation. *Methods of Inf Med* 2007;**46 (5)**: 574-579.

77. Gralla O, Haas F, Knoll N, Hadzidiakos D, Tullmann M, Romer A, Deger S, Ebeling V, Lein M, Wille A, Rehberg B, Loening SA, Roigas J. Fast-track surgery in laparoscopic radical prostatectomy: basic principles. *World Journal of Urology* 2007;**25**(2): 185-191.

78. Guiahi M, Goldman KN, McElhinney MM, Olson CG, Guiahi M, Goldman KN, McElhinney MM, Olson CG. Improving hysterosalpingogram confirmatory test follow-up after Essure hysteroscopic sterilization. *Contraception* 2010;**81**(6): 520-524.

79. Halm MA. Collaborative care: improving patient outcomes in cardiovascular surgery. *Progress in Cardiovascular Nursing* 1997;**12**(2): 15-23.

80. Hammer J, Harling H, Wille-Jorgensen P. Implementation of the scientific evidence into daily practice--example from fast-track colonic cancer surgery. *Colorectal Disease* 2008;**10**(6): 593-598.

81. Harvey CV. Collaborative development of a standardized order form for orthopaedics. *Orthopaedic Nursing* 1990;**9**(1): 34-37.

82. Haynes AB, Weiser TG, Berry WR, Lipsitz SR, Breizat AH, Dellinger EP, Herbosa T, Joseph S, Kibatala PL, Lapitan MC, Merry AF, Moorthy K, Reznick RK, Taylor B, Gawande AA, Safe Surgery Saves Lives Study G, Haynes AB, Weiser TG, Berry WR, Lipsitz SR, Breizat A-HS, Dellinger EP, Herbosa T, Joseph S, Kibatala PL, Lapitan MCM, Merry AF, Moorthy K, Reznick RK, Taylor B, Gawande AA. A surgical safety checklist to reduce morbidity and mortality in a global population. *N Engl J Med* 2009;**360**(5): 491-499.

83. Healy W. Impact of a clinical pathway and implant standardization on total hip arthroplasty. *J Arthroplasty* 1998;**13**: 266-276.

84. Hedrick TL, Turrentine FE, Smith RL, McElearney ST, Evans HL, Pruett TL, Sawyer RG. Single-institutional experience with the surgical infection prevention project in intra-abdominal surgery. *Surg Infect* 2007;**8**(4): 425-435.

85. Henderson A, Prendergast E. Algorithmic nursing protocol to provide optimum care post combined kidney/pancreas transplantation. *Edtna Erca J* 1999;**25**(4): 21-23.

86. Henrickson SE, Wadhera RK, Elbardissi AW, Wiegmann DA, Sundt TM, 3rd, Henrickson SE, Wadhera RK, Elbardissi AW, Wiegmann DA, Sundt TM, 3rd. Development and pilot evaluation of a preoperative briefing protocol for cardiovascular surgery. *J Am Coll Surg* 2009;**208**(6): 1115-1123.

87. Holtzman J. The effects of clinical pathways for renal transplant on patient outcomes and length of stay *Med Care* 1998;**36**: 826-834.

88. Huber TS, Carlton LM, Harward TR, Russin MM, Phillips PT, Nalli BJ, Flynn TC, Seeger JM. Impact of a clinical pathway for elective infrarenal aortic reconstructions. *Ann Surg* 1998;**227**(5): 691-699.

89. Husbands JM, Weber RS, Karpati RL, Weinstein GS, Chalian AA, Goldberg AN, Thaler ER, Wolf PF. Clinical care pathways: Decreasing resource utilization in head and neck surgical patients. *Otolaryngol Head Neck Surg* 1999;**121 (6)**: 755-759.

90. Husted H, Holm G. Fast track in total hip and knee arthroplasty--experiences from Hvidovre University Hospital, Denmark.[Erratum appears in Injury. 2007 Oct;38(10):1224]. *Injury* 2006;**37**(5).

91. Husted H, Otte KS, Kristensen BB, Orsnes T, Kehlet H. Readmissions after fast-track hip and knee arthroplasty. *Archives of Orthopaedic & Trauma Surgery* 2010;**130**(9): 1185-1191.

92. Husted H, Hansen HC, Holm G, Bach-Dal C, Rud K, Andersen KL, Kehlet H. What determines length of stay after total hip and knee arthroplasty? A nationwide study in Denmark. *Arch Orthop Trauma Surg* 2010;**130 (2)**: 263-268.

93. Irizarry JM, Graham MH, Cordts PR. Use of a critical pathway to move laparoscopic cholecystectomy to the ambulatory surgery arena. *Mil Med* 1999;**164**(7): 531-534.

94. Isozaki LF, Fahndrick J. Clinical pathways--a perioperative application. *AORN Journal* 1998;**67**(2): 374-392.

95. Jakobsen DH, Sonne E, Andreasen J, Kehlet H. Convalescence after colonic surgery with fast-track vs conventional care. *Colorectal Disease* 2006(of Publication: Oct 2006): 8 (8) (pp 683-687), 2006.

96. Jiang K, Cheng L, Wang JJ, Li JS, Nie J. Fast track clinical pathway implications in esophagogastrectomy. *World Journal of Gastroenterology* 2009;**15**(4): 496-501.

97. Joh YG, Lindsetmo RO, Stulberg J, Obias V, Champagne B, Delaney CP. Standardized postoperative pathway: accelerating recovery after ileostomy closure. *Dis Colon Rectum* 2008;**51**(12): 1786-1789.

98. Johnston G, Ekert L, Pally E. Surgical site signing and "time out": issues of compliance or complacence. *J Bone Joint Surg Am* 2577;**91**(11): 2577-2580.

99. Jottard KJ, van Berlo C, Jeuken L, Dejong C, group E, Jottard KJC. Changes in outcome during implementation of a fast-track colonic surgery project in a university-affiliated general teaching hospital: advantages reached with ERAS (Enhanced Recovery After Surgery project) over a 1-year period. *Dig Surg* 2008;**25**(5): 335-338.

100. Kallenbach AM, Rosenblum CJ. Carotid endarterectomy: creating the pathway to 1-day stay. *Crit Care Nurse* 2000;**20 (4)**: 23-26, 28-29, 31-36.

101. Kao LS, Lew DF, Doyle PD, Carrick MM, Jordan VS, Thomas EJ, Lally KP, Kao LS, Lew DF, Doyle PD, Carrick MM, Jordan VS, Thomas EJ, Lally KP. A tale of 2 hospitals: a staggered cohort study of targeted interventions to improve compliance with antibiotic prophylaxis guidelines. *Surgery* 2010;**148**(2): 255-262.

102. Kariv Y, Delaney CP, Senagore AJ, Manilich EA, Hammel JP, Church JM, Ravas J, Fazio VW. Clinical outcomes and cost analysis of a "fast track" postoperative care pathway for ileal pouch-anal anastomosis: a case control study. *Dis Colon Rectum* 2007;**50**(2): 137-146.

103. Kawahara H, Yanagisawa S, Kashiwagi H, Hirai K, Yamazaki Y, Yanaga K. Implementation of a clinical pathway for laparoscopic colorectal surgery. *Int Surg* 2005;**90 (3)**: 144-147.

104. Kehlet H, Mogensen, T. Hospital stay of two-days after open sigmoidectomy

with a multi-modal rehabilitation programme. *Br J Surg* 1999;**86**: 227–230.

105. Kennedy EP, Rosato EL, Sauter PK, Rosenberg LM, Doria C, Marino IR, Chojnacki KA, Berger AC, Yeo CJ. Initiation of a Critical Pathway for Pancreaticoduodenectomy at an Academic Institution-the First Step in Multidisciplinary Team Building. *J Am Coll Surg* 2007;**204 (5)**: 917-923.

106. Khoo CK, Vickery CJ, Forsyth N, Vinall NS, Eyre-Brook IA. A prospective randomized controlled trial of multimodal perioperative management protocol in patients undergoing elective colorectal resection for cancer. *Ann Surg* 2007;**245 (6)**: 867-872.

107. Knight N, Aucar J, Knight N, Aucar J. Use of an anatomic marking form as an alternative to the Universal Protocol for Preventing Wrong Site, Wrong Procedure and Wrong Person Surgery. *Am J Surg* 2010;**200**(6): 803-807; discussion 807-809.

108. Koval KJ, Chen AL, Aharonoff GB, Egol KA, Zuckerman JD. Clinical pathway for hip fractures in the elderly: the Hospital for Joint Diseases experience. *Clin Orthop Relat Res* 2004;**425**: 72-81.

109. Kremer M, Ulrich A, Buchler MW, Uhl W. Fast-track surgery: the Heidelberg experience. [Review] [25 refs]. *Recent Results in Cancer Research* 2005;**165**: 14-20.

110. Krenzer ME. Unplugging the mystery of carotid endarterectomy patient care. *Crit Care Nurs Clin North Am* 1999;**11**(2): 189-208.

111. Kulkarni RP, Ituarte PH, Gunderson D, Yeh MW, Kulkarni RP, Ituarte PHG, Gunderson D, Yeh MW. Clinical pathways improve hospital resource use in endocrine surgery. *J Am Coll Surg* 2011;**212**(1): 35-41.

112. Larson DW, Batdorf NJ, Touzios JG, Cima RR, Chua HK, Pemberton JH, Dozois EJ. A fast-track recovery protocol improves outcomes in elective laparoscopic colectomy for diverticulitis. *Journal of the American College of Surgeons* 2010;**211**(4): 485-489.

113. Lazar HL, Fitzgerald CA, Ahmad T, Bao Y, Colton T, Shapira OM, Shemin RJ. Early discharge after coronary artery bypass graft surgery: are patients really going home earlier? *Journal of Thoracic & Cardiovascular Surgery* 2001;**121**(5): 943-950.

114. Leibman BD, Dillioglugil, O., Abbas, F., Tanli, S., Kattan, M W., Scardino, P T. Impact of a clinical pathway for radical retropubic prostatectomy. *Urology* 1998;**52**(1): 94-99.

115. Lemmens L, van Zelm R, Vanhaecht K, Kerkkamp H. Systematic review: indicators to evaluate effectiveness of clinical pathways for gastrointestinal surgery. [Review] [42 refs]. *Journal of Evaluation in Clinical Practice* 2008;**14**(5): 880-887.

116. Lemmens L, van Zelm R, Borel Rinkes I, van Hillegersberg R, Kerkkamp H. Clinical and organizational content of clinical pathways for digestive surgery: a systematic review. [Review] [44 refs]. *Digestive Surgery* 2009;**26**(2): 91-99.

117. Ley A. Fast tracking in cardiac surgery: the St. Francis experience. *Nurs Case Manag* 1998;**3** (4): 155-159.

118. Lindsetmo RO, Champagne B, Delaney CP. Laparoscopic rectal resections and fast-track surgery: what can be expected? *American Journal of Surgery* 2009;**197**(3): 408-412.

119. Lingard L, Espin S, Rubin B, Whyte S, Colmenares M, Baker GR, Doran D, Grober E, Orser B, Bohnen J, Reznick R. Getting teams to talk: Development and pilot implementation of a checklist to promote interprofessional communication in the OR. *Qual Saf Health Care* 2005;**14 (5)**: 340-346.

120. Lingard L, Whyte S, Espin S, Baker GR, Orser B, Doran D, Lingard L, Whyte S, Espin S, Baker GR, Orser B, Doran D. Towards safer interprofessional communication: constructing a model of "utility" from preoperative team briefings. *J Interprof Care* 2006;**20**(5): 471-483.

121. Lingard L, Regehr, G., Orser, B., Reznick, R., Baker, GR., Doran, D., Espin, S., Bohnen, J., Whyte, S. Evaluation of a preoperative checklist and team briefing among surgeons, nurses, and anesthesiologists to reduce failures in communication. *Archives of Surgery* 2008;**143**(1): 12-17.

122. Liu XX, Jiang ZW, Wang ZM, Li JS. Multimodal optimization of surgical care shows beneficial outcome in gastrectomy surgery. *Journal of Parenteral and Enteral Nutrition* 2010(of Publication: May 2010): 34 (33) (pp 313-321), 2010.

123. Mabrey JD, Toohey, J S., Armstrong, D A., Lavery, L., Wammack, L A. Clinical pathway management of total knee arthroplasty. *Clin Orthop Relat Res* 1997;**345**: 125-133.

124. MacKenzie M, Waterman M. Utilization of a clinical pathway in the care of the ambulatory cataract surgical patient. *Insight* 1995;**20**(2): 6-11.

125. Maessen J, Dejong CH, Hausel J, Nygren J, Lassen K, Andersen J, Kessels AG, Revhaug A, Kehlet H, Ljungqvist O, Fearon KC, von Meyenfeldt MF. A protocol is not enough to implement an enhanced recovery programme for colorectal resection. *Br J Surg* 2007;**94**(2): 224-231.

126. Mandal K, Dodds SG, Hildreth A, Fraser SG, Steel DH, Mandal K, Dodds SG, Hildreth A, Fraser SG, Steel DHW. Comparative study of first-day postoperative cataract review methods. *J Cataract Refract Surg* 2004;**30**(9): 1966-1971.

127. Markey DW, McGowan, J., Hanks, J B. The effect of clinical pathway implementation on total hospital costs for thyroidectomy and parathyroidectomy patients. *Am Surg* 2000;**66**(6): 533-538.

128. Maruyama R, Miyake, T., Kojo, M., Aoki, Y., Suemitsu, R., Okamoto, T., Wataya, H., Ichinose, Y. Establishment of a clinical pathway as an effective tool to reduce hospitalization and charges after video-assisted thoracoscopic pulmonary resection. *Jpn J Thorac Cardiovasc Surg* 2006;**54**(9): 387-390.

129. Marx C, Rasmussen T, Hjort Jakobsen D, Ottosen C, Lundvall L, Ottesen B, Callesen T, Kehlet H. The effect of accelerated rehabilitation on recovery after surgery for ovarian malignancy. *Acta Obstetricia et Gynecologica Scandinavica* 2006(of Publication: Mar 2006): 85 (84) (pp 488-492), 2006.

130. Marzen-Groller KD, Tremblay SM, Kaszuba J, Girodo V, Swavely D, Moyer B, Bartman K, Carraher W, Wilson E, Marzen-Groller KD, Tremblay SM, Kaszuba J, Girodo V, Swavely D, Moyer B, Bartman K, Carraher W, Wilson E. Testing the effectiveness of the Amputee Mobility Protocol: a pilot study. *J Vasc Nurs* 2008;**26**(3): 74-81.

131. Matsumoto A, Kanda K, Shigematsu H. Development and implementation of a critical pathway for abdominal aortic aneurysms in Japan. *J Vasc Nurs* 2002;**20**(1): 14-21.

132. Maxam-Moore VA, Goedecke RS. The development of an early extubation algorithm for patients after cardiac surgery. *Heart Lung* 1996;**25**(1): 61-68.

133. McAchran SE, Goldman HB. Contemporary Length of Stay and Resource Utilization When Using a Fast-track Regimen for Mid-urethral Sling Surgery. *Urology* 2009;**74**(3): 531-534.

134. McLellan RA, Bell DG, Rendon RA. Effective analgesia and decreased length of stay for patients undergoing radical prostatectomy: Effectiveness of a clinical pathway. *Can J Urol* 2006;**13**(5): 3244-3249.

135. Melbert RB, Kimmins MH, Isler JT, Billingham RP, Lawton D, Salvadalena G, Cortezzo M, Rowbotham R. Use of a critical pathway for colon resections. *J Gastrointest Surg* 2002;**6**(5): 745-752.

136. Mikulaninec CE. An amputee critical path. *Journal of Vascular Nursing* 1992;**10**(2): 6-9.

137. Mo J, Oh H, Ahn Y, Seo W, Mo J, Oh H, Ahn Y, Seo W. Implementation of a clinical pathway in primary spontaneous pneumothorax patients treated by wedge resection surgery. *Clin Nurse Spec* 2010;**24**(6): 295-303.

138. Muehling BM, Halter GL, Schelzig H, Meierhenrich R, Steffen P, Sunder-Plassmann L, Orend KH. Reduction of postoperative pulmonary complications after lung surgery using a fast track clinical pathway. *Eur J Cardiothorac Surg* 2008;**34**(1): 174-180.

139. Muehling BM, Halter G, Lang G, Schelzig H, Steffen P, Wagner F, Meierhenrich R, Sunder-Plassmann L, Orend KH. Prospective randomized controlled trial to evaluate "fast-track" elective open infrarenal aneurysm repair. *Langenbecks Archives of Surgery* 2008;**393**(3): 281-287.

140. Muehling B, Schelzig H, Steffen P, Meierhenrich R, Sunder-Plassmann L, Orend KH. A prospective randomized trial comparing traditional and fast-track patient care in elective open infrarenal aneurysm repair. *World Journal of Surgery* 2009;**33**(3): 577-585.

141. Muller S, Zalunardo MP, Hubner M, Clavien PA, Demartines N, Zurich Fast Track Study G. A fast-track program reduces complications and length of hospital stay after open colonic surgery. *Gastroenterology* 2009;**136**(3): 842-847.

142. Munitiz V, Martinez-de-Haro LF, Ortiz A, Ruiz-de-Angulo D, Pastor P, Parrilla P. Effectiveness of a written clinical pathway for enhanced recovery after transthoracic (Ivor Lewis) oesophagectomy. *Br J Surg* 2010;**97**(5): 714-718.

143. Murphy MA, Richards T, Atkinson C, Perkins J, Hands LJ. Fast track open aortic surgery: reduced postoperative stay with a goal directed pathway. *Eur J Vasc Endovasc Surg* 2007;**34**(3): 274-278.

144. Musser DJ, Calligaro KD, Dougherty MJ, Raviola CA, DeLaurentis DA. Safety and cost-efficiency of 24-hour hospitalization for carotid endarterectomy. *Ann Vasc Surg* 1996;**10**(2): 143-146.

145. Nagpal K, Vats A, Lamb B, Ashrafian H, Sevdalis N, Vincent C, Moorthy K, Nagpal K, Vats A, Lamb B, Ashrafian H, Sevdalis N, Vincent C, Moorthy K. Information transfer and communication in surgery: a systematic review. *Ann Surg* 2010;**252**(2): 225-239.

146. Naughton C, Cheek L, O'Hara K. Rapid recovery following cardiac surgery: a nursing perspective. *Br J Nurs* 2005;**14**(4): 214-219.

147. Neily J, Mills PD, Young-Xu Y, Carney BT, West P, Berger DH, Mazzia LM, Paull DE, Bagian JP, Neily J, Mills PD, Young-Xu Y, Carney BT, West P, Berger DH, Mazzia LM, Paull DE, Bagian JP. Association between implementation of a medical team training program and surgical mortality. *JAMA* 2010;**304**(15): 1693-1700.

148. Nilsson L, Lindberget O, Gupta A, Vegfors M. Implementing a pre-operative checklist to increase patient safety: a 1-year follow-up of personnel attitudes. *Acta Anaesthesiol Scand* 2010;**54**(2): 176-182.

149. Nuelle DG, Mann K. Minimal incision protocols for anesthesia, pain management, and physical therapy with standard incisions in hip and knee arthroplasties: the effect on early outcomes. *J Arthroplasty* 2007;**22**(1): 20-25.

150. Nygren J, Hausel J, Kehlet H, Revhaug A, Lassen K, Dejong C, Andersen J, von Meyenfeldt M, Ljungqvist O, Fearon KC. A comparison in five European Centres of case mix, clinical management and outcomes following either conventional or fast-track perioperative care in colorectal surgery. *Clinical Nutrition* 2005;**24**(3): 455-461.

151. Okita A, Yamashita M, Abe K, Nagai C, Matsumoto A, Akehi M, Yamashita R, Ishida N, Seike M, Yokota S, Umekawa N, Matsumoto Y, Kishimoto Y, Okazaki A, Komori E, Sawada S, Takashima S. Variance analysis of a clinical pathway of video-assisted single lobectomy for lung cancer. *Surg Today* 2009;**39** (2): 104-109.

152. Oldmeadow LB, McBurney H, Robertson VJ, Kimmel L, Elliott B. Targeted postoperative care improves discharge outcome after hip or knee arthroplasty. *Arch Phys Med Rehabil* 1424;**85**(9): 1424-1427.

153. Ottesen M, Sorensen M, Rasmussen Y, Smidt-Jensen S, Kehlet H, Ottesen B. Fast track vaginal surgery. *Acta Obstetricia et Gynecologica Scandinavica* 2002;**81**(2): 138-146.

154. Paige JT, Aaron DL, Yang T, Howell DS, Hilton CW, Cohn I, Jr., Chauvin SW, Paige JT, Aaron DL, Yang T, Howell DS, Hilton CW, Cohn I, Jr., Chauvin SW. Implementation of a preoperative briefing protocol improves accuracy of teamwork assessment in the operating room. *Am Surg* 2008;**74**(9): 817-823.

155. Paige JT, Aaron DL, Yang T, Howell DS, Chauvin SW. Improved operating room teamwork via SAFETY prep: A rural community hospital's experience. *World J Surg* 2009;**33 (6)**: 1181-1187.

156. Paone G, Higgins RS, Havstad SL, Silverman NA. Does age limit the effectiveness of clinical pathways after coronary artery bypass graft surgery? *Circulation* 1998;**98**(19 Suppl): 10.

157. Paull DE, Mazzia LM, Wood SD, Theis MS, Robinson LD, Carney B, Neily J, Mills PD, Bagian JP, Paull DE, Mazzia LM, Wood SD, Theis MS, Robinson LD, Carney B, Neily J, Mills PD, Bagian JP. Briefing guide study: preoperative briefing and postoperative debriefing checklists in the Veterans Health Administration medical team training program. *Am J Surg* 2010;**200**(5): 620-623.

158. Pearson S, Moraw, I., Maddern, G J. Clinical pathway management of total knee arthroplasty: a retrospective comparative study. *Aust N Z J Surg* 2000;**70**(5): 351-354.

159. Pearson SD, Kleefield SF, Soukop JR, Cook EF, Lee TH. Critical pathways intervention to reduce length of hospital stay. *Am J Med* 2001;**110**(3): 175-180.

160. Pedersen SJ, Borgbjerg FM, Schousboe B, Pedersen BD, Jorgensen HL, Duus BR, Lauritzen JB, Hip Fracture Group of Bispebjerg H. A comprehensive hip fracture program reduces complication rates and mortality. *J Am Geriatr Soc* 2008;**56**(10): 1831-1838.

161. Petersen MK, Andersen NT, Soballe K. Self-reported functional outcome after primary total hip replacement treated with two different periopera-tive regimes: a follow-up study involving 61 patients. *Acta Orthopaedica* 2008;**79**(2): 160-167.

162. Pitt HA, Murray, K P., Bowman, H M., Coleman, J., Gordon, T A., Yeo, C J., Lillemoe, K D., Cameron, J L. Clinical pathway implementation improves outcomes for complex biliary surgery. *Surgery* 1999;**126**(4): 751-756.

163. Podore PC, Throop EB. Infrarenal aortic surgery with a 3-day hospital stay: A report on success with a clinical pathway. *J Vasc Surg* 1999;**29**(5): 787-792.

164. Polle SW, Wind J, Fuhring JW, Hofland J, Gouma DJ, Bemelman WA. Implementation of a fast-track perioperative care program: what are the difficulties? *Digestive Surgery* 2007;**24**(6): 441-449.

165. Porter GA, Pisters, P W., Mansyur, C., Bisanz, A., Reyna, K., Stanford, P., Lee, J E., Evans, D B. Cost and utilization impact of a clinical pathway for patients undergoing pancreaticoduodenectomy. *Annals of Surgical Oncology* 2000;**7**(7): 484-489.

166. Pritts TA, Nussbaum MS, Flesch LV, Fegelman EJ, Parikh AA, Fischer JE. Implementation of a clinical pathway decreases length of stay and cost for bowel resection. *Annals of Surgery* 1999;**230**(5): 728-733.

167. Prouty A, Cooper M, Thomas P, Christensen J, Strong C, Bowie L, Oermann MH, Prouty A, Cooper M, Thomas P, Christensen J, Strong C, Bowie L, Oermann MH. Multidisciplinary patient education for total joint replacement surgery patients. *Orthop Nurs* 2006;**25**(4): 257-261; quiz 262-253.

168. Pruthi RS, Chun J, Richman M. Reducing time to oral diet and hospital discharge in patients undergoing radical cystectomy using a perioperative care plan. *Urology* 2003;**62**(4): 661-665.

169. Pruthi RS, Nielsen M, Smith A, Nix J, Schultz H, Wallen EM. Fast track program in patients undergoing radical cystectomy: results in 362 consecutive patients. *Journal of the American College of Surgeons* 2010;**210**(1): 93-99.

170. Quigley RL, Reitknecht FL. A coronary artery bypass 'fast-track' protocol is practical and realistic in a rural environment. *Annals of Thoracic Surgery* 1997(of Publication: Sep 1997): 64 (63) (pp 706-709), 1997.

171. Raue W, Haase O, Junghans T, Scharfenberg M, Muller JM, Schwenk W. 'Fast-track' multimodal rehabilitation program improves outcome after laparoscopic sigmoidectomy: a controlled prospective evaluation. *Surgical Endoscopy* 2004;**18**(10): 1463-1468.

172. Recart A, Duchene D, White PF, Thomas T, Johnson DB, Cadeddu JA. Efficacy and safety of fast-track recovery strategy for patients undergoing laparoscopic nephrectomy. *Journal of Endourology* 2005;**19**(10): 1165-1169.

173. Renkawitz T, Rieder T, Handel M, Koller M, Drescher J, Bonnlaender G, Grifka J. Comparison of two accelerated clinical pathways--after total knee replacement how fast can we really go? *Clin Rehabil* 2010;**24**(3): 230-239.

174. Riddle MM, Dunstan JL, Castanis JL. A rapid recovery program for cardiac surgery patients. *Am J Crit Care* 1996;**5**(2): 152-159.

175. Rogers JP, Novchich TM, Pearce GL, Johnston JS, Burton HG, 3rd, Groh MA. Port-access cardiac surgery protocols and early outcomes. *Critical Care Nursing Clinics of North America* 1998;**10**(1): 61-73.

176. Rogers SN, Naylor R, Potter L, Magennis P. Three years' experience of collaborative care pathways on a maxillofacial ward. *Br J Oral Maxillofac Surg* 2000;**38**(2): 132-137.

177. Ronellenfitsch U, Rossner E, Jakob J, Post S, Hohenberger P, Schwarzbach M, Ronellenfitsch U, Rossner E, Jakob J, Post S, Hohenberger P, Schwarzbach M. Clinical Pathways in surgery: should we introduce them into clinical routine? A review article. *Langenbecks Arch Surg* 2008;**393**(4): 449-457.

178. Rouse AD, Tripp BL, Shipley S, Pories W, Cunningham P, MacDonald K, Jr. Meeting the challenge of managed care through clinical pathways for bariatric surgery. *Obesity Surgery* 1998;**8**(5): 530-534.

179. Rusynko B, Perry-Ewald J, Rusynko B, Perry-Ewald J. Keeping patients safe--procedure and site verification and preprocedure pause. *Aorn J* 2004;**79**(4): 787-793.

180. Sakallaris BR, Halpin LS, Knapp M, Sheridan MJ. Same-day transfer of patients to the cardiac telemetry unit after surgery: the Rapid after Bypass Back into Telemetry (RABBIT) program. *Critical Care Nurse* 2000;**20**(2): 50-55.

181. Santoso U, Iau, P T C., Lim, J., Koh, C S L., Pang, Y T. The mastectomy clinical pathway: what has it achieved?. *Ann Acad Med Singapore* 2002;**31**(4): 440-445.

182. Scatizzi M, Kroning KC, Boddi V, De Prizio M, Feroci F. Fast-track surgery after laparoscopic colorectal surgery: is it feasible in a general surgery unit? *Surgery* 2010;**147**(2): 219-226.

183. Scharfenberg M, Raue W, Junghans T, Schwenk W. "Fast-track" rehabilitation after colonic surgery in elderly patients--is it feasible? *International Journal of Colorectal Disease* 2007;**22**(12): 1469-1474.

184. Schneider M, Kawahara I, Ballantyne G, McAuley C, MacGregor K, Garvie R, McKenzie A, MacDonald D, Breusch SJ. Predictive factors influencing fast track rehabilitation following primary total hip and knee arthroplasty. *Archives of Orthopaedic and Trauma Surgery* 2009(of Publication: December 2009): 129 (112) (pp 1585-1591), 2009.

185. Schwarzbach M, Bonninghoff R, Harrer K, Weiss J, Denz C, Schnulle P, Birck R, Post S, Ronellenfitsch U. Effects of a clinical pathway on quality of care in kidney transplantation: a non-randomized clinical trial. *Langenbecks Arch Surg* 2010;**395**(1): 11-17.

186. Schwenk W, Neudecker J, Raue W, Haase O, Muller JM. "Fast-track" rehabilitation after rectal cancer resection. *International Journal of Colorectal Disease* 2006;**21**(6): 547-553.

187. Schwenk W, Gunther N, Wendling P, Schmid M, Probst W, Kipfmuller K, Rumstadt B, Walz MK, Engemann R, Junghans T, Fast-track" Colon IIQAG. "Fast-track" rehabilitation for elective colonic surgery in Germany--prospective observational data from a multi-centre quality assurance programme. *Int J Colorectal Dis* 2008;**23**(1): 93-99.

188. Serclova Z, Dytrych P, Marvan J, Nova K, Hankeova Z, Ryska O, Slegrova Z, Buresova L, Travnikova L, Antos F. Fast-track in open intestinal surgery: Prospective randomized study (Clinical Trials Gov Identifier no. NCT00123456). *Clinical Nutrition* 2009(of Publication: December 2009): 28 (26) (pp 618-624), 2009.

189. Sherman D, Matthews TW, Lampe H, LeBlanc S. Laryngectomy clinical pathway: development and review. *J Otolaryngol* 2001;**30**(2): 115-120.

190. Sladek ML, Swenson KK, Ritz LJ, Schroeder LM. A critical pathway for patients undergoing one-day breast cancer surgery. *Clin J Oncol Nurs* 1999;**3 (3)**: 99-106.

191. So JBY, Lim ZL, Lin HA, Ti TK. Reduction of hospital stay and cost after the implementation of a clinical pathway for radical gastrectomy for gastric cancer. *Gastric Cancer* 2008;**11 (2)**: 81-85.

192. Soria V, Pellicer E, Flores B, Carrasco M, Candel MF, Aguayo JL. Evaluation of the clinical pathway for laparoscopic cholecystectomy. *Am Surg* 2005;**71 (1)**: 40-45.

193. Spanjersberg WR, Reurings J, Keus F, van Laarhoven CJ. Fast track surgery versus conventional recovery strategies for colorectal surgery. [Review]. *Cochrane Database of Systematic Reviews* 2011;**2**.

194. Stanford JR, Swaney-Berghoff L, Recht KE, Orsagh-Yentis DK. Improved cardiac surgical outcomes with use of total quality management. *J Clin Outcomes Manag* 2009;**16 (9)**: 405-409.

195. Stephen AE, Berger DL. Shortened length of stay and hospital cost reduction with implementation of an accelerated clinical care pathway after elective colon resection. *Surgery* 2003;**133**(3): 277-282.

196. Stidham KR, Roberson JB. Implementation of a clinical pathway in management of the postoperative vestibular schwannoma patient. *Laryngoscope* 2001;**111**(11 Pt 1): 1938-1943.

197. Strong AG, Sneed NV. Clinical evaluation of a critical path for coronary artery bypass surgery patients. *Prog Cardiovasc Nurs* 1991;**6**(1): 29-37.

198. Tan JJ, Foo AY, Cheong DM. Colorectal clinical pathways: a method of improving clinical outcome? *Asian J Surg* 2005;**28**(4): 252-256.

199. Teeuwen PHE, Bleichrodt RP, Strik C, Groenewoud JJM, Brinkert W, van Laarhoven CJHM, van Goor H, Bremers AJA. Enhanced Recovery After Surgery (ERAS) versus conventional postoperative care in colorectal surgery. *Journal of Gastrointestinal Surgery* 2009(of Publication: January 2009): 14 (11) (pp 88-95), 2009.

200. Thomas K. Clinical pathway for hip and knee arthroplasty. *Physiotherapy* 2003;**89** (10): 603-609.

201. Tomaszek SC, Cassivi SD, Allen MS, Shen KR, Nichols FC, 3rd, Deschamps C, Wigle DA, Tomaszek SC, Cassivi SD, Allen MS, Shen KR, Nichols FC, 3rd, Deschamps C, Wigle DA. An alternative postoperative pathway reduces length of hospitalisation following oesophagectomy. *Eur J Cardiothoracic Surg* 2010;**37**(4): 807-813.

202. Topal B, Peeters G, Verbert A, Penninckx F. Outpatient laparoscopic cholecystectomy: clinical pathway implementation is efficient and cost effective and increases hospital bed capacity. *Surg Endosc* 1142;**21**(7): 1142-1146.

203. Toraman F, Evrenkaya S, Yuce M, Goksel O, Karabulut H, Alhan C. Fast-track recovery in noncoronary cardiac surgery patients. *Heart Surgery Forum* 2005;**8**(1).

204. Tromp F, Dulmen S, Weert J. Interdisciplinary preoperative patient education in cardiac surgery. *J Adv Nurs* 2004;**47**(2): 212-222.

205. Trussell J, Gerkin R, Coates B, Brandenberger J, Tibi P, Keuth J, Montefour K, Salisbury H, Ferrara J. Impact of a patient care pathway protocol on surgical site infection rates in cardiothoracic surgery patients. *Am J Surg* 2008;**196**(6): 883-889.

206. Uchiyama K, Takifuji, K., Tani, M., Onishi, H., Yamaue, H. Effectiveness of the clinical pathway to decrease length of stay and cost for laparoscopic surgery. *Surg Endosc* 2002;**16**(11): 1594-1597.

207. Ueda K, Kaneda Y, Sakano H, Tanaka T, Li TS, Hamano K. Obstacles for shortening hospitalization after video-assisted pulmonary resection for lung cancer. *Ann Thorac Surg* 2003;**76**(6): 1816-1820.

208. van Dam RM, Hendry PO, Coolsen MM, Bemelmans MH, Lassen K, Revhaug A, Fearon KC, Garden OJ, Dejong CH, Enhanced Recovery After Surgery G. Initial experience with a multimodal enhanced recovery programme in patients undergoing liver resection. *British Journal of Surgery* 2008;**95**(8): 969-975.

209. Van Herck P, Vanhaecht K, Deneckere S, Bellemans J, Panella M, Barbieri A, Sermeus W. Key interventions and outcomes in joint arthroplasty clinical pathways: A systematic review. *J Eval Clin Pract* 2010;**16 (1)**: 39-49.

210. Varadhan KK, Lobo DN, Ljungqvist O, Varadhan KK, Lobo DN, Ljungqvist O. Enhanced recovery after surgery: the future of improving surgical care. *Crit Care Clin* 2010;**26**(3): 527-547.

211. Varadhan KK, Neal KR, Dejong CHC, Fearon KCH, Ljungqvist O, Lobo DN. The enhanced recovery after surgery (ERAS) pathway for patients undergoing major elective open colorectal surgery: A meta-analysis of randomized controlled trials. *Clinical Nutrition* 2010(of Publication: August 2010): 29 (24) (pp 434-440), 2010.

212. Veltman R, Loppnow N. Improving care for patients having abdominal hysterectomy. *Hosp Case Manag* 1999;**7**(8): 139-142.

213. Wadhera RK, Parker SH, Burkhart HM, Greason KL, Neal JR, Levenick KM, Wiegmann DA, Sundt TM, 3rd, Wadhera RK, Parker SH, Burkhart HM, Greason KL, Neal JR, Levenick KM, Wiegmann DA, Sundt TM, 3rd. Is the "sterile cockpit" concept applicable to cardiovascular surgery critical intervals or critical events? The impact of protocol-driven communication during cardiopulmonary bypass. *J Thorac Cardiovasc Surg* 2010;**139**(2): 312-319.

214. Wainwright T, Middleton R. An orthopaedic enhanced recovery pathway. *Curr Anaesth Crit Care* 2010;**21 (3)**: 114-120.

215. Walsh MD, Barry M, Scott TE, Lamorte WW, Menzoian JO. The role of a nurse case manager in implementing a critical pathway for infrainguinal bypass surgery. *Jt Comm J Qual Improv* 2001;**27**(4): 230-238.

216. Walter FL, Bass N, Bock G, Markel DC. Success of clinical pathways for total joint arthroplasty in a community hospital. *Clin Ortho Relat Res* 2007;**(457)**: 133-137.

217. Wang D, Kong Y, Zhong B, Zhou X, Zhou Y. Fast-track surgery improves postoperative recovery in patients with gastric cancer: a randomized comparison with conventional postoperative care. *Journal of Gastrointestinal Surgery* 2010;**14**(4): 620-627.

218. Watters CL, Moran WP. Hip fractures--a joint effort. *Orthop Nurs* 2006;**25**(3): 157-165.

219. Webster TM, Baumgartner R, Sprunger JK, Baldwin DD, McDougall EM, Herrell SD, Webster TM, Baumgartner R, Sprunger JK, Baldwin DD, McDougall EM, Herrell SD. A clinical pathway for laparoscopic pyeloplasty decreases length of stay. *J Urol* 2005;**173**(6): 2081-2084.

220. Wehberg K, E. , Jackson D, Walters J, Redmond B, Todd IJC, Ogburn NL, Leonard S. Fast track minimally invasive transmyocardial revascularization. *Innovations: Technology and Techniques in Cardiothoracic and Vascular Surgery* 2009(of Publication: July-August 2009): 4 (4) (pp 217-220), 2009.

221. Weiser TG, Haynes AB, Dziekan G, Berry WR, Lipsitz SR, Gawande AA, Safe Surgery Saves Lives I, Study G. Effect of a 19-item surgical safety checklist during urgent operations in a global patient population. *Ann Surg* 2010;**251**(5): 976-980.

222. White MM, Gupta M, Utman SA, Dhillon B, Utman SAK. Importance of side marking in ophthalmic surgery. *Surgeon* 2009;**7**(2): 82-85.

223. Wichmann MW, Roth M, Jauch KW, Bruns CJ. A prospective clinical feasibility study for multimodal "fast track" rehabilitation in elective pancreatic cancer surgery. *Rozhl Chir* 2006;**85**(4): 169-175.

224. Wichmann MW, Eben R, Angele MK, Brandenburg F, Goetz AE, Jauch KW. Fast-track rehabilitation in elective colorectal surgery patients: a prospective clinical and immunological single-centre study. *ANZ Journal of Surgery* 2007;**77**(7): 502-507.

225. Wind J, Polle SW, Fung Kon Jin PH, Dejong CH, von Meyenfeldt MF, Ubbink DT, Gouma DJ, Bemelman WA, Laparoscopy and/or Fast Track Multimodal Management Versus Standard Care Study G, Enhanced Recovery after Surgery G. Systematic review of enhanced recovery programmes in colonic surgery. [Review] [29 refs]. *British Journal of Surgery* 2006;**93**(7): 800-809.

226. Wright CD, Wain, J C., Grillo, H C., Moncure, A C., Macaluso, S M., Mathisen, D J. Pulmonary lobectomy patient care pathway: a model to control cost and maintain quality. *Ann Thorac Surg* 1997;**64**(2): 299-302.

227. Yanagi K, Sasajima K, Miyamoto M, Suzuki S, Yokoyama T, Maruyama H, Matsutani T, Arima Y, Uchida E, Tajiri T. Evaluation of the clinical pathway for laparoscopic cholecystectomy and simulation of short-term hospitalization. *J Nippon Med Sch* 2007;**74 (6)**: 409-413.

228. Yanatori M, Tomita S, Miura Y, Ueno Y. Feasibility of the fast-track recovery program after cardiac surgery in Japan. *General Thoracic and Cardiovascular Surgery* 2007(of Publication: Nov 2007): 55 (11) (pp 445-449), 2007.

229. Yang CT, Chen HH, Hou SM. Patient safely in Taiwan: A survey on orthopedic surgeons. *J Formos Med Assoc* 2007;**106** (3): 212-216.

230. Zargar-Shoshtari K, Connolly AB, Israel LH, Hill AG. Fast-track surgery may reduce complications following major colonic surgery. *Dis Colon Rectum* 2008;**51**(11): 1633-1640.

231. Zargar-Shoshtari K, Paddison JS, Booth RJ, Hill AG. A prospective study on the influence of a fast-track program on postoperative fatigue and functional recovery after major colonic surgery. *J Surg Res* 2009;**154**(2): 330-335.

232. Zargar-Shoshtari K, Hill AG. Fast-track open colectomy is possible in a New Zealand public hospital. *New Zealand Medical Journal* 2008;**121**(1275): 33-36.
